# Supplementary material for: Tuft cell acetylcholine is released into the gut lumen as an effector and regulator of type 2 immunity, and directly targets helminth parasites
Source: Immunity. Author manuscript; Available in PMC 2026 Sep 4. (PMC7619444; doi:10.1016/j.immuni.2024.04.018)
Supplement: Supplementary file 1 [file EMS217793-supplement-S1.pdf]

Immunity, Volume *57*

Supplemental information

Tuft cell acetylcholine is released into the gut lumen to promote anti-helminth immunity

Marième Ndjim, Imène Gasmi, Fabien Herbert, Charlène Joséphine, Julie Bas, Ali Lamrani, Nathalie Coutry, Sylvain Henry, Valérie S. Zimmermann, Valérie Dardalhon, Marta Campillo Poveda, Evgenia Turtoi, Steeve Thirard, Luc Forichon, Alicia Giordano, Claire Ciancia, Zeinab Homayed, Julie Pannequin, Collette Britton, Eileen Devaney, Tom N. McNeilly, Sylvie Berrard, Andrei Turtoi, Rick M. Maizels, François Gerbe, and Philippe Jay

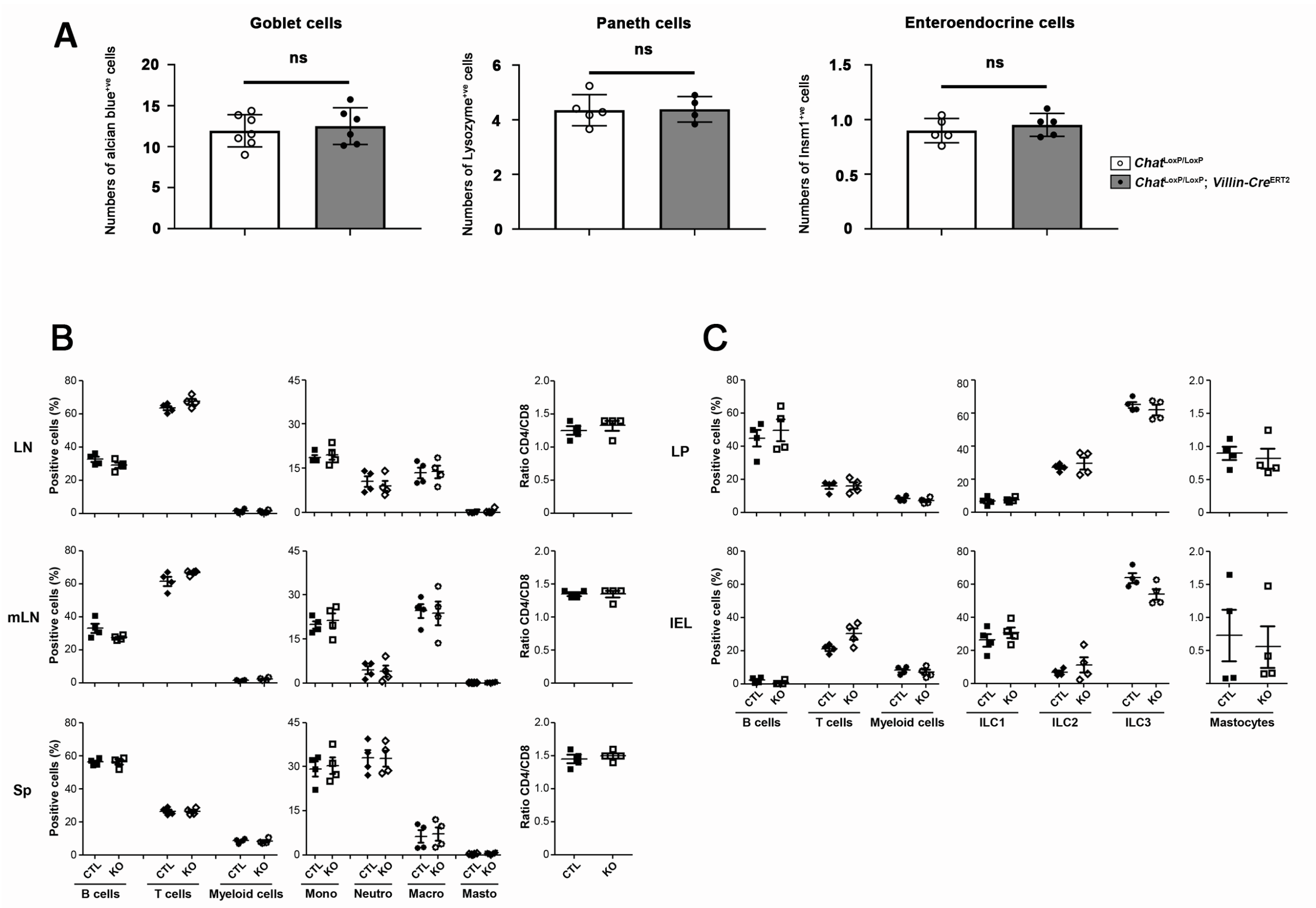

Figure S1: Intestinal epithelial ChAT deficiency does not cause drastic changes in intestinal epithelium cellular composition and does not impact the immune landscape in the intestine and in distant lymphoid organs, related to Figure 2. (A) Goblet, Paneth and enteroendocrine cells were counted in naive mice, 10 days after recombination of the *chat* locus. Alcian blue staining was used to highlight goblet cells and IHC against lysozyme and Insm1, to reveal Paneth and enteroendocrine cells, respectively. Data represent means ± SD of the biological replicates (n ranging from 4 to 7, ns = p > 0.05 after a Student T test). **(B)** The repartition of immune cells in control (*Chat*$^{LoxP/LoxP}$) and *Chat*-deficient (*Chat*$^{LoxP/LoxP}$;*Villin-Cre*^{ERT2}) mice was monitored by flow cytometry in lymph nodes (LN), mesenteric lymph nodes (mLN) and spleens (Sp). The presence of T (TCRβ+) including CD4/CD8 ratio, B (CD19+), total myeloid (CD11b+) cells as well monocytes (mono, CD11b+Ly6C+Ly6G-), neutrophils (neutro, CD11b+Ly6C+Ly6G+), macrophages (macro, CD11b+F4/80+), mastocytes (CD11b+CD117+FcεRIa+) was assessed by staining with fluorochrome-tagged antibodies. Each point represents one mouse and means ± SD are shown. (n = 4 mice per genotype). **(C)** Immune lineage cells in the *lamina propria* (LP) and intra-epithelial lymphocyte (IEL) of control *(Chat*$^{LoxP/LoxP}$) and *Chat*-deficient (*Chat*$^{LoxP/LoxP}$;*Villin-Cre*^{ERT2}) mice were monitored by flow cytometry after tissue dissociation. The percentage of T, B and myeloid cells were assessed within the CD45+ haematopoietic gate, and ILC subsets within the CD45+CD19-CD3-CD11b-CD127+ population. Representative dot plots are presented. Quantification of immune cells are shown; each point represents a mouse as means ± SD (n = 4 mice per genotype). Statistical analyses were performed using a Mann Whitney test and no significant differences were detected for all cell types between *Chat*$^{LoxP/LoxP}$ and *Chat*$^{LoxP/LoxP}$*;Villin-Cre*^{ERT2} mice.

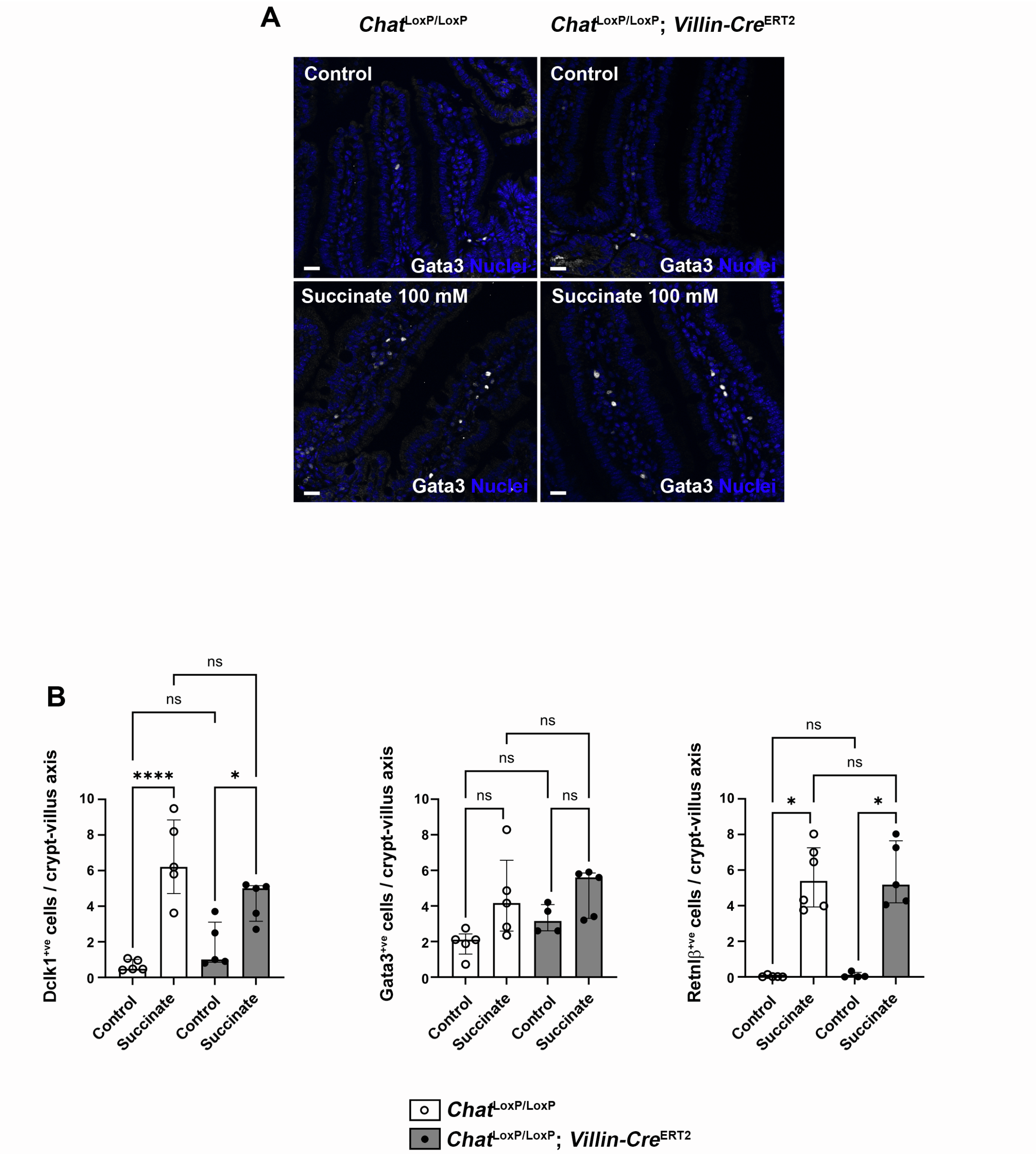

Figure S2: ChAT-deficient mice are able to mount a type-2 immune response after a succinate-mediated tuft cell activation, related to Figure 4. (A) Representative Gata3 staining (grey) by IHC for both *Chat*$^{LoxP/LoxP}$ and *Chat*$^{LoxP/LoxP}$;*Villin-Cre*^{ERT2} mice, treated or not with 100 mM succinate for one week (scales: 10µm) **(B).** Quantification of type-2 immune response parameters in *Chat*$^{LoxP/LoxP}$ and *Chat*$^{LoxP/LoxP}$;*Villin-Cre*^{ERT2} mice, treated or not with 100 mM succinate for one week, as assessed by counting Dclk1-expressing tuft cells (n=5 for each group), Gata3-expressing ILC2/Th2 cells (n=4 and 5), and Resistin-like beta-expressing goblet cells (n=4 and 6). Data represent median ± interquartile of the biological replicates (n= 4 to 6 for each condition, ns = $p > 0.05$, * = $p < 0.05$, **** = $p < 0.0001$ after Kruskal-Wallis analyse).

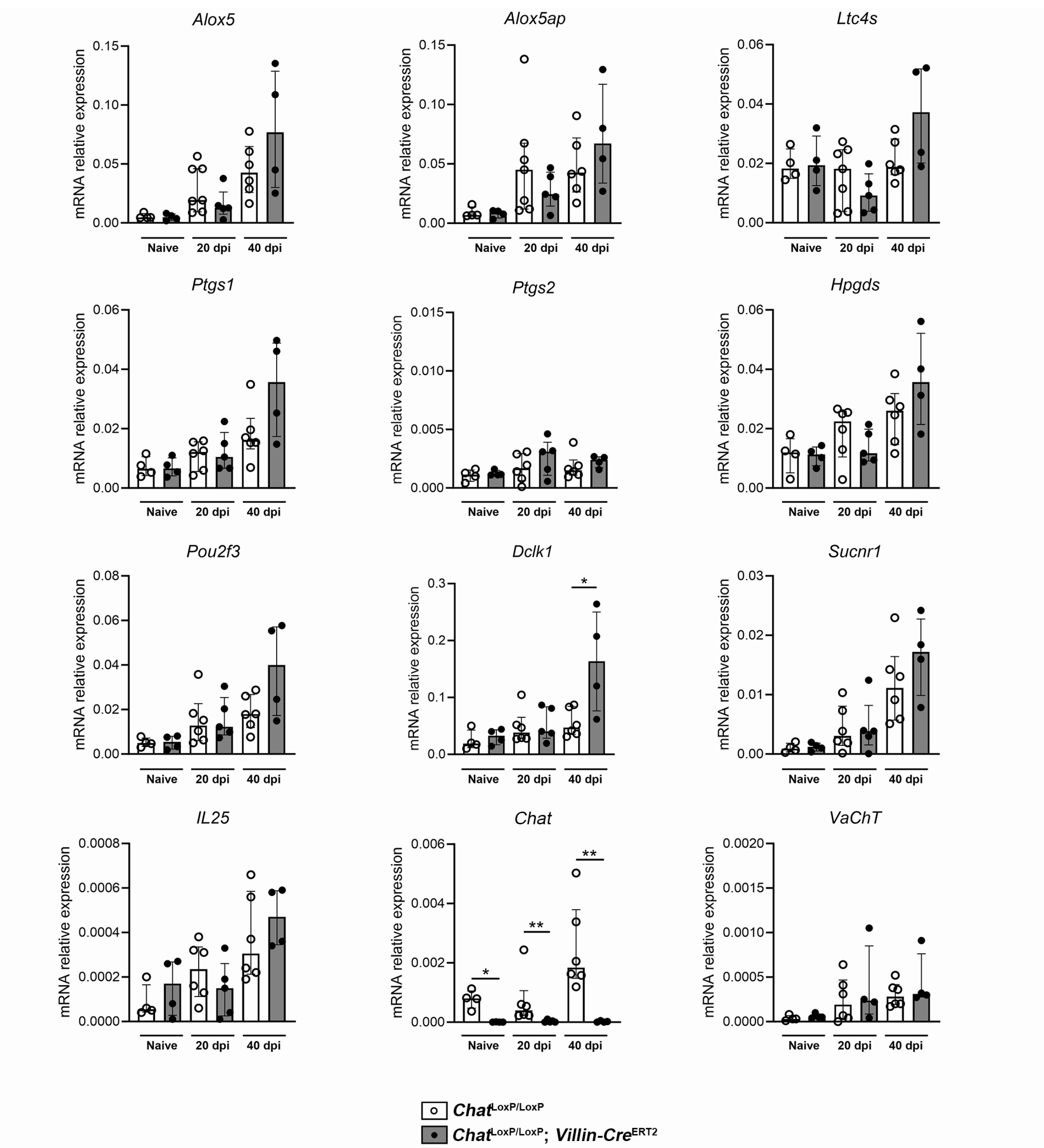

Figure S3: Expression levels of transcripts related to leukotrienes and prostanoids biosynthesis pathways, tuft cells markers and alarmin function, and ACh synthesis and vesicular transport, related to Figure 4. PCR was performed on mRNA extracted from enriched epithelial cell suspensions recovered from 4 to 7 tamoxifen-treated naive and *H. polygyrus*-infected *Chat*$^{LoxP/LoxP}$ control (white bar and circles) or *Chat*$^{LoxP/LoxP}$;*Villin-Cre*^{ERT2} mice (grey bar and circles), 20 and 40 days post-infection. Data represent median ± interquartile of the biological replicates, * = $p < 0.05$, ** = $p < 0.01$, after a Mann Whitney test.

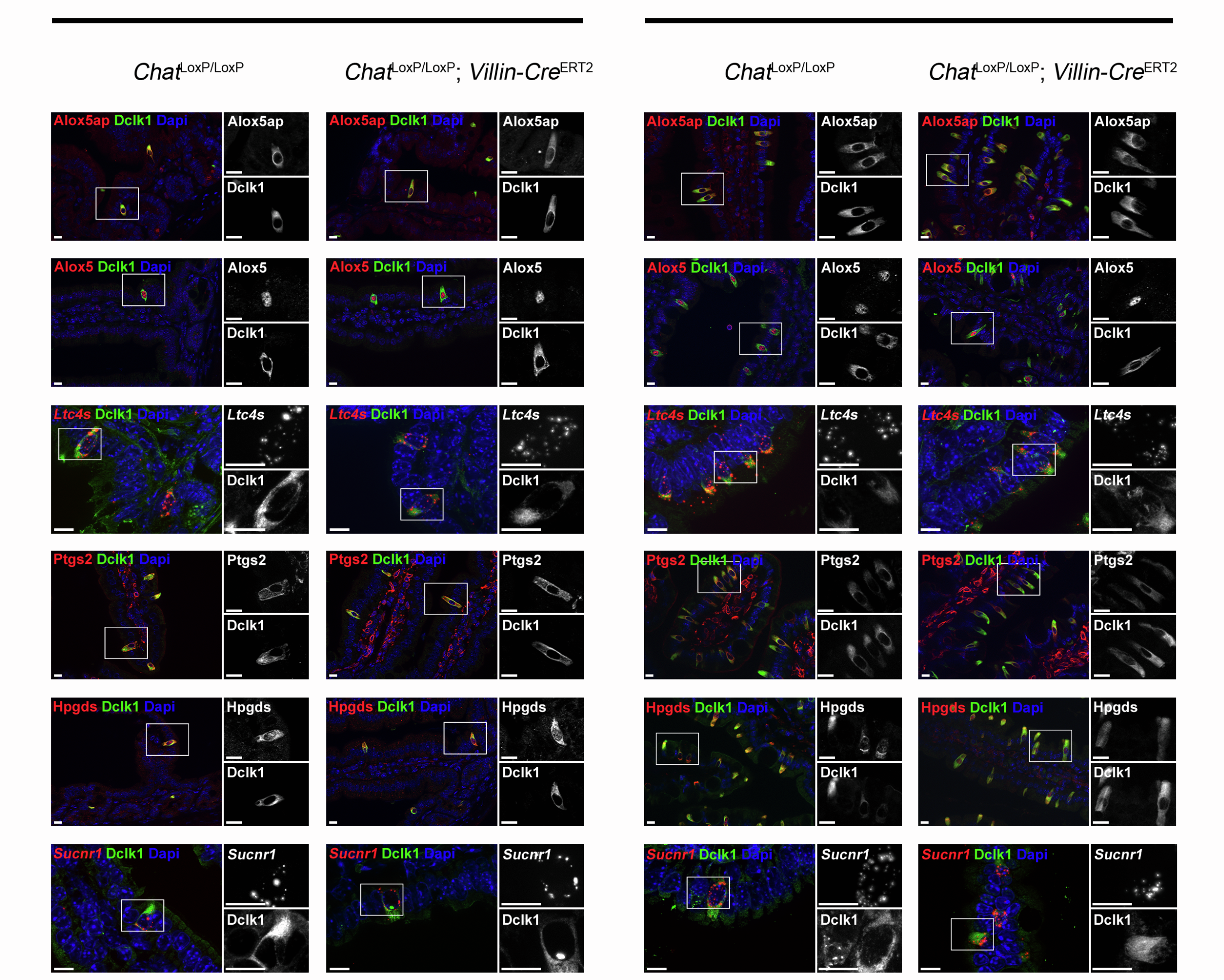

Figure S4: ChAT deficiency does not impair expression of tuft cell specific, type-2 immune response-related proteins, related to Figure 4. Representative double IHC, or RNAscope combined with IHC, for the tuft cell marker Dclk1 (green on overlays), leukotriene C4 biosynthesis (Alox, Alox5ap and *Ltc4s*), PGD_2 biosynthesis (Ptgs2, Hpgds), and succinate receptor (*Sucnr1*), in tamoxifen-treated naive and *H. polygyrus*-infected (20 days post-infection) *Chat*$^{LoxP/LoxP}$ and *Chat*$^{LoxP/LoxP}$;*Villin-Cre*^{ERT2} mice (n=3 for each condition). Scales: 10µm.

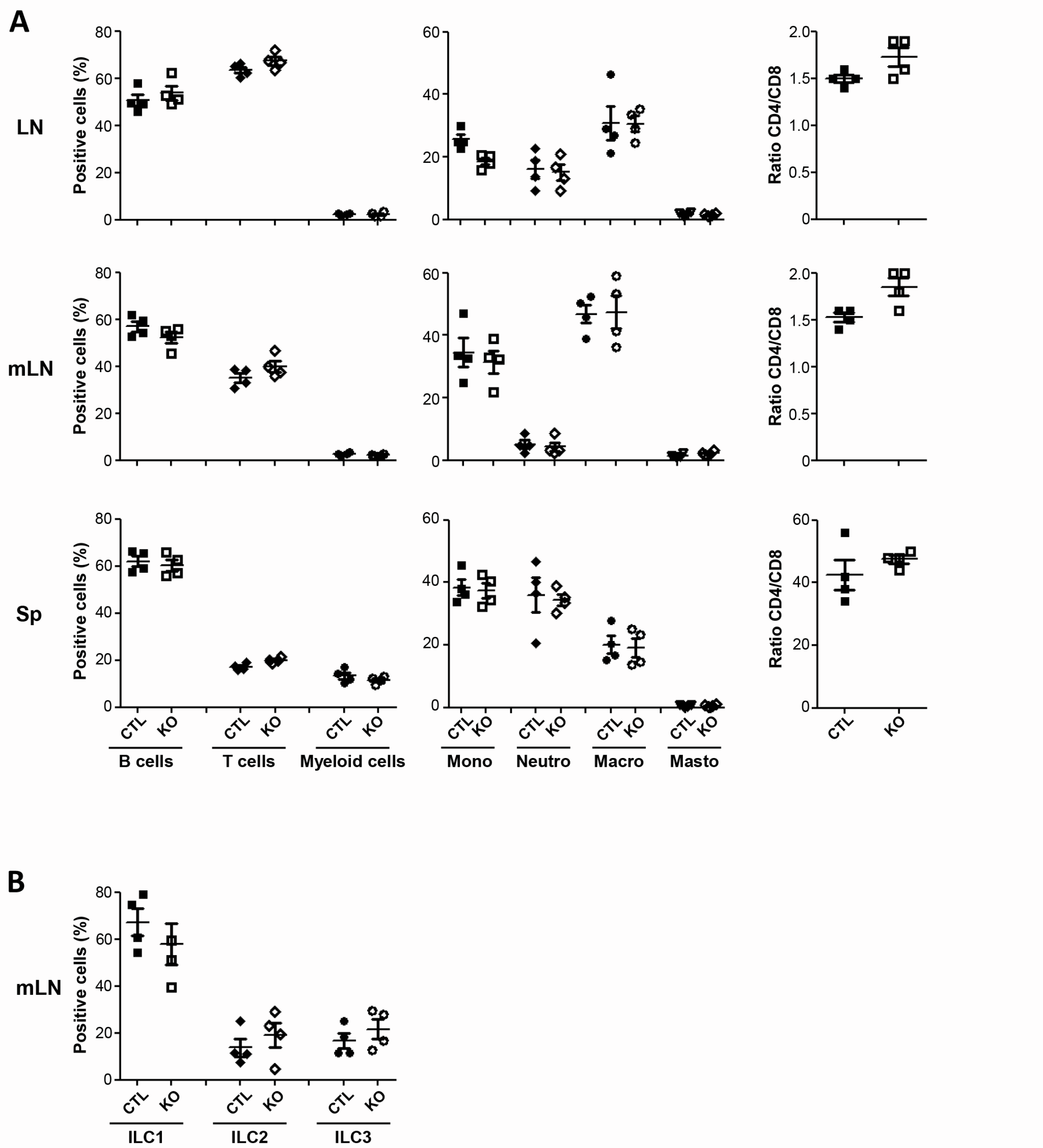

Figure S5: Epithelial ChAT deficiency does not impact the immune landscape within the intestine and in distant lymphoid organs during infections, related to Figure 4. (A) The repartition of immune cells in wild-type (*Chat*$^{LoxP/LoxP}$) and ChAT-deficient (*Chat*$^{LoxP/LoxP}$;*Villin-Cre*^{ERT2}) mice was monitored by flow cytometry in lymph nodes (LN), mesenteric lymph nodes (mLN) and spleens (Sp) following *H. polygyrus* infections. The presence of T (TCRβ+) including CD4/CD8 ratio, B (CD19+), total myeloid (CD11b+) cells as well as monocytes (mono, CD11b+Ly6C+Ly6G-), neutrophils (neutro, CD11b+Ly6C+Ly6G+), macrophages (macro, CD11b+F4/80+), mastocytes (CD11b+CD117+FcεRIa+) was assessed by staining with fluorochrome-tagged antibodies. Each point represents one mouse and means ± SD are shown. (n = 4 mice per genotype). **(B)** Immune lineage cells in the mesenteric lymph nodes (mLN) of *H. polygyrus*-infected control (*Chat*$^{LoxP/LoxP}$) and ChAT-deficient (*Chat*$^{LoxP/LoxP}$;*Villin-Cre*^{ERT2}) mice were monitored by flow cytometry after tissue dissociation. The percentage of ILC subsets were assessed within the CD45+CD19-CD3-CD11b-CD127+ population. Representative dot plots are presented. Quantification of immune cells are shown; each point represents a mouse as means ± SD (n = 4 mice per genotype). Statistical analyses were performed using a Mann Whitney -test and no significant differences were detected for all cell types between *Chat*$^{LoxP/LoxP}$ and *Chat*$^{LoxP/LoxP}$;*Villin-Cre*^{ERT2} mice.

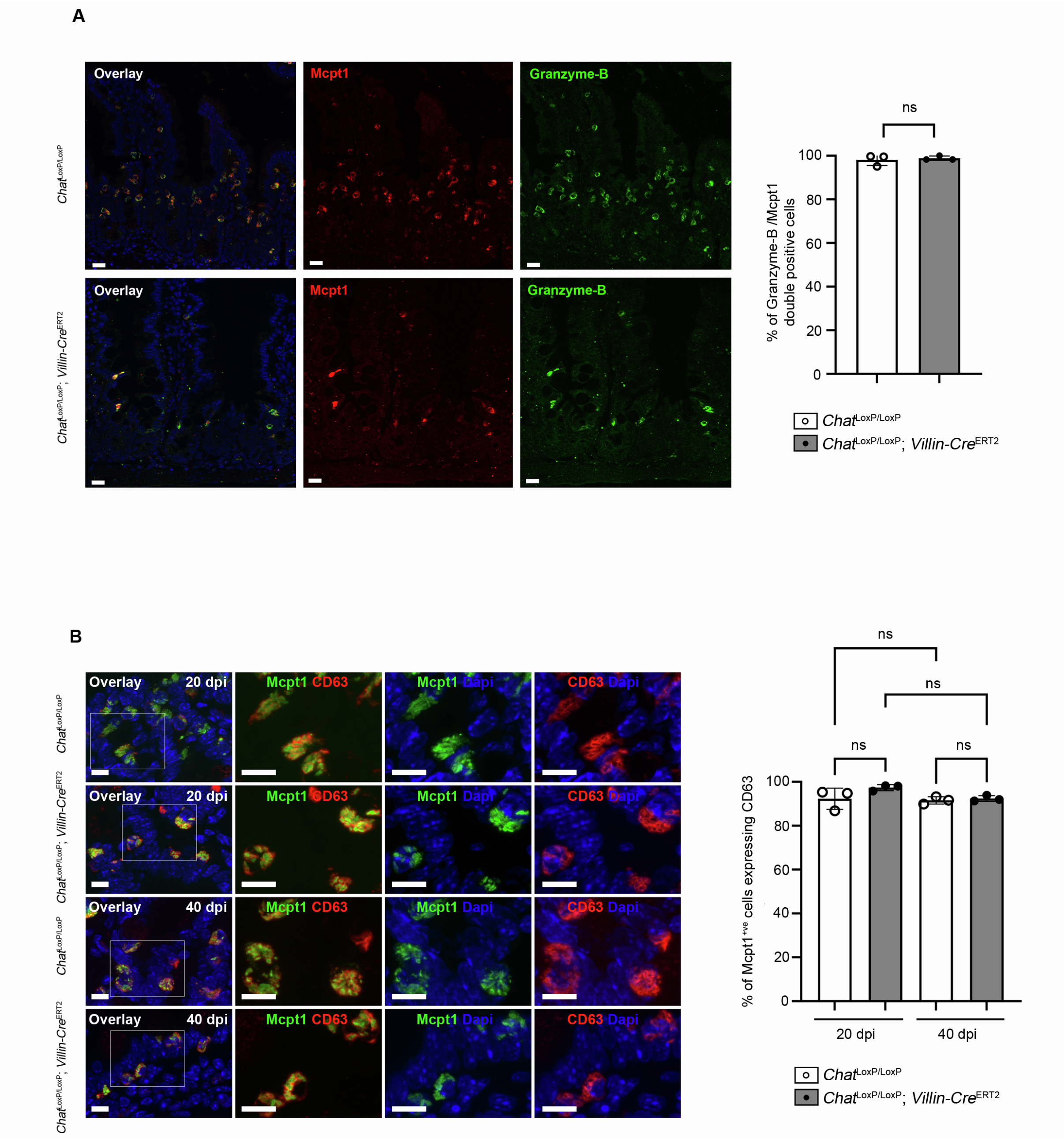

Figure S6: ChAT deficiency does not impair mast cell marker expression and activation, related to Figure 5. (A) Representative double IHC for Mcpt1 (red) and Granzyme-B (green), from 20 dpi mice. The graph represents the percentage of co-expression between these two markers. Data represent median ± interquartile of the biological replicates (for a total 318 and 783 counted mast cells in n=3 mice from both *Chat*$^{LoxP/LoxP}$ and *Chat*$^{LoxP/LoxP}$;*Villin-Cre*^{ERT2} genotypes, ns = p > 0.05 after a Mann-Whitney test). **(B)** Representative immunostaining for Mcpt1 (green) and CD63 (red) in *Chat*$^{LoxP/LoxP}$ and *Chat*$^{LoxP/LoxP}$;*Villin-Cre*^{ERT2} mice, at 20 and 40 dpi with *H. polygyrus*. (Scales: 10µm). Graph showing the percentage of co-expression between these two markers. Data represent means ± SD of the biological replicates, for a total of 469 and 324 counted mast (20 dpi), and 239 and 305 counted mast cells (40 dpi), in 3 mice of both *Chat*$^{LoxP/LoxP}$ and *Chat*$^{LoxP/LoxP}$;*Villin-Cre*^{ERT2} genotypes, ns = p > 0.05 after ANOVA analyse.

Mouse ID	Recovered volume of intestinal content (µl)
1	20
2	18
3	8
4	9
5	13
6	18
**Mean**	**14.33**
**SD**	**4.64**

Figure S7: Estimation of small intestinal luminal content volumes prior to surgeries, related to Figure 6. The table shows recovered volumes of small intestinal contents prior to surgeries. Data were acquired from 6 mice, means and SD are shown.
