# Supplementary material for: Tuft cell acetylcholine is released into the gut lumen as an effector and regulator of type 2 immunity, and directly targets helminth parasites
Source: Immunity. Author manuscript; Available in PMC 2026 Sep 4. (PMC7619444; doi:10.1016/j.immuni.2024.04.018)
Supplement: Supplementary file 2 [file EMS217793-supplement-S8.pdf]

Article

Immunity

# Tuft cell acetylcholine is released into the gut lumen to promote anti-helminth immunity

## Graphical abstract

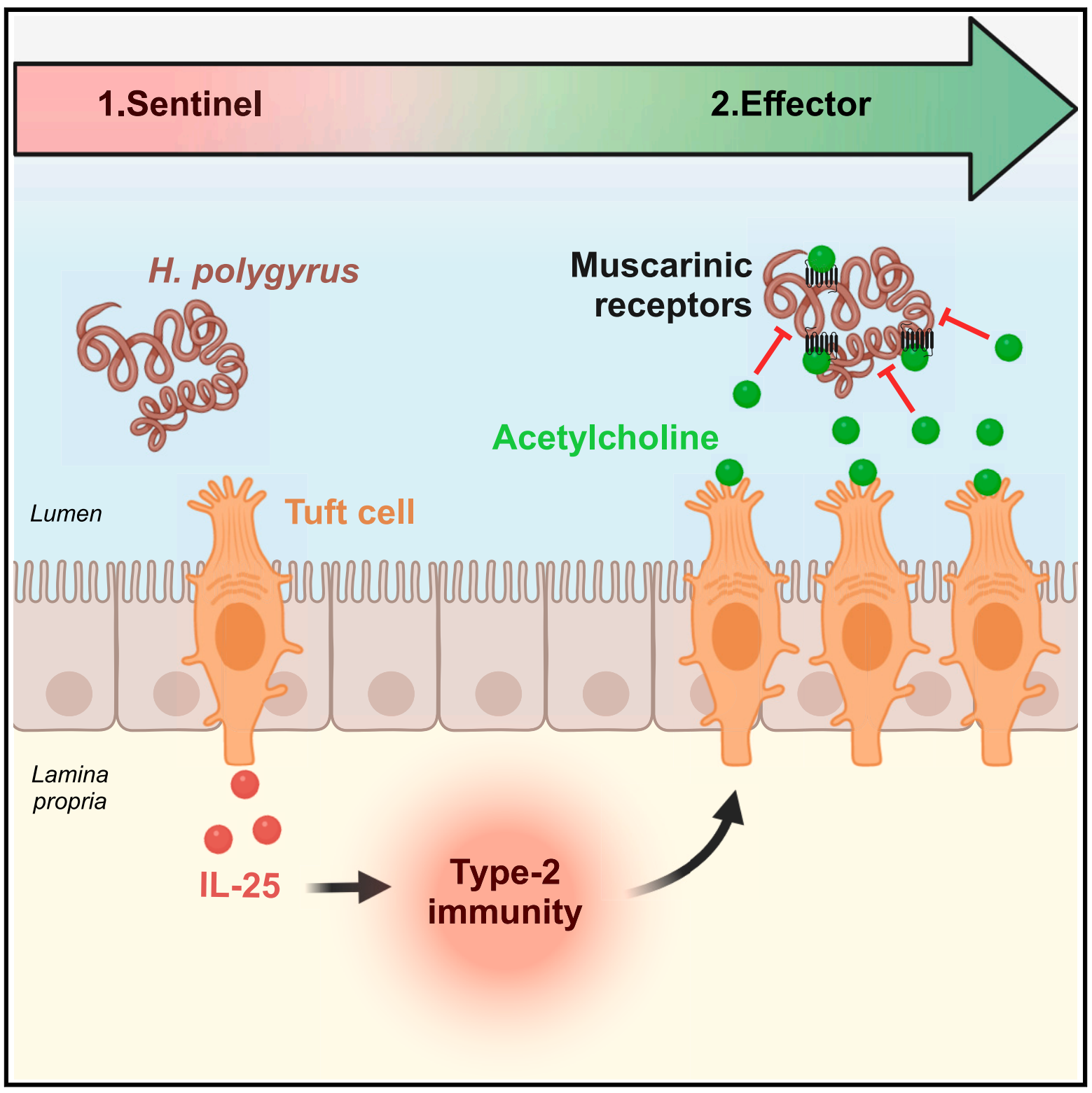


## Highlights

- Tuft cells play both sentinel and effector roles in type 2 immune responses
- Tuft cell acetylcholine production increases during helminth infections
- Tuft cells release acetylcholine into the gut lumen during type 2 immune responses
- Acetylcloline inhibits helminth fecundity through worm muscarinic receptors

## Authors

Marième Ndjim, Imène Gasmi, Fabien Herbert, ..., Rick M. Maizels, François Gerbe, Philippe Jay

## Correspondence

francois.gerbe@igf.cnrs.fr (F.G.), philippe.jay@igf.cnrs.fr (P.J.)

## In brief

Intestinal tuft cells are known as sentinels capable of initiating type 2 immune responses upon parasite infections. Ndjim et al. now report a direct effector function for intestinal tuft cells during such immune responses by increasing their acetylcholine biosynthesis and releasing it into the gut lumen. Helminth fecundity is directly inhibited by acetylcholine, acting through worm muscarinic receptors.

Ndjim et al., 2024, Immunity *57*, 1260–1273
June 11, 2024 © 2024 The Authors. Published by Elsevier Inc.
https://doi.org/10.1016/j.immuni.2024.04.018

CellPress

CellPress
OPEN ACCESS

Immunity

Article

# Tuft cell acetylcholine is released into the gut lumen to promote anti-helminth immunity

Marième Ndjim,[1,9] Imène Gasmi,[1,9] Fabien Herbert,[1,9] Charlène Joséphine,[1] Julie Bas,[1] Ali Lamrani,[1] Nathalie Coutry,[1] Sylvain Henry,[2] Valérie S. Zimmermann,[3] Valérie Dardalhon,[3] Marta Campillo Poveda,[4] Evgenia Turtoi,[2] Steeve Thirard,[1] Luc Forichon,[1] Alicia Giordano,[1] Claire Ciancia,[4] Zeinab Homayed,[1] Julie Pannequin,[1] Collette Britton,[5] Eileen Devaney,[5] Tom N. McNeilly,[6] Sylvie Berrard,[7] Andrei Turtoi,[2,8] Rick M. Maizels,[4] François Gerbe,[1,*] and Philippe Jay[1,10,*]

[1]Institute of Functional Genomics (IGF), University of Montpellier, CNRS, Inserm, Montpellier, France
[2]Montpellier Alliance for Metabolomics and Metabolism Analysis, Platform for Translational Oncometabolomics (PLATON), Biocampus, CNRS, INSERM, Université de Montpellier, Montpellier, France
[3]Institut de Génétique Moléculaire de Montpellier, University of Montpellier, CNRS, Montpellier, France
[4]Centre for Parasitology, School of Infection and Immunity, University of Glasgow, Glasgow G12 8TA, UK
[5]School of Biodiversity, One Health and Veterinary Medicine, University of Glasgow, Glasgow, UK
[6]Disease Control Department, Moredun Research Institute, Penicuik, UK
[7]University Paris Cité, Inserm, NeuroDiderot, Paris, France
[8]Cancer Research Institute of Montpellier (IRCM), University of Montpellier, Inserm, Montpellier, France
[9]These authors contributed equally
[10]Lead contact
*Correspondence: francois.gerbe@igf.cnrs.fr (F.G.), philippe.jay@igf.cnrs.fr (P.J.)
https://doi.org/10.1016/j.immuni.2024.04.018

## SUMMARY

Upon parasitic helminth infection, activated intestinal tuft cells secrete interleukin-25 (IL-25), which initiates a type 2 immune response during which *lamina propria* type 2 innate lymphoid cells (ILC2s) produce IL-13. This causes epithelial remodeling, including tuft cell hyperplasia, the function of which is unknown. We identified a cholinergic effector function of tuft cells, which are the only epithelial cells that expressed choline acetyltransferase (ChAT). During parasite infection, mice with epithelial-specific deletion of ChAT had increased worm burden, fitness, and fecal egg counts, even though type 2 immune responses were comparable. Mechanistically, IL-13-amplified tuft cells release acetylcholine (ACh) into the gut lumen. Finally, we demonstrated a direct effect of ACh on worms, which reduced their fecundity via helminth-expressed muscarinic ACh receptors. Thus, tuft cells are sentinels in naive mice, and their amplification upon helminth infection provides an additional type 2 immune response effector function.

## INTRODUCTION

Tuft cells are a cellular subset mostly found in digestive and respiratory epithelia and play critical roles in mucosal host defense. In the intestinal epithelium, tuft cells are primarily known for their critical sentinel function during parasite infections. The presence of helminth or protozoa in the gut triggers tuft cell secretion of the alarmin cytokine interleukin-25 (IL-25) that initiates a type 2 immune response.[1–3] Such a response is principally orchestrated by type 2 innate lymphoid cells (ILC2s) through the secretion of type 2 cytokines such as IL-4, IL-5, and IL-13. A critical aspect of IL-4 and IL-13 function is the profound remodeling they cause in the intestinal epithelium. This includes amplification of the mucus-producing goblet cells and the tuft cell lineages, as well as resistin-like beta (Retnlβ) ectopic expression by small intestinal goblet cells, in which it is usually absent.[4] While Retnlβ directly interferes with worm physiology,[4–6] increased mucus production and smooth muscle hypercontractility, also known as the "weep and sweep" response, facilitate worm expulsion.[7] In contrast, the physiological role of the dramatic increase in tuft cell numbers during type 2 immune responses is not yet understood. Increased IL-25 production following tuft cell lineage amplification is thought to lead to a more efficient type 2 immune response,[1,3] but alternative tuft cell functions also need to be considered. Firstly, we noted that this amplification occurred downstream of the action of type 2 cytokines on epithelial cells; secondly, we noted that worm expulsion was substantially more delayed by the absence of tuft cells[1] as compared to IL-25 deficiency alone. These points strongly suggest that, in addition to their alarm function,[8] tuft cells are required not only as initiators but rather as an integral effector component of the type 2 immune response.

The acetylcholine (ACh) neurotransmitter, biosynthesized by the choline acetyltransferase (ChAT) enzyme, regulates a variety

 © 2024 The Authors. Published by Elsevier Inc.
This is an open access article under the CC BY license (http://creativecommons.org/licenses/by/4.0/).

CellPress
OPEN ACCESS

of neuronal and non-neuronal physiological functions.[9] Studies with *Chat* reporter mice revealed the presence of cholinergic epithelial cells in various tissues including the trachea,[10] urethra,[11] gastrointestinal and biliary tracts,[12] and thymus.[13] These cells were identified as tuft (also called brush or solitary chemosensory) cells in the airway, where their actual ACh release was demonstrated.[14] In the nasal cavity, cholinergic tuft cells regulate breathing and inflammation in the presence of irritants[15]; in the trachea, they control breathing reflexes[16] and muco-ciliary clearance in response to bacterial quorum sensing molecules.[17] Additionally, urethral tuft cells control micturition reflexes through cholinergic signaling to viscerosensory neurons in response to exogenous bitter compounds.[11] In contrast, the function of intestinal ChAT-expressing cells remains unknown.

Interestingly, some commonly used drugs against intestinal helminths, such as levamisole and pyrantel, are cholinergic agonists. They target the worm ACh receptors (AChRs), causing spastic paralysis, which facilitates worm expulsion[18] and suggests potential direct effects of host ACh on parasites. In addition, available ACh for signaling results from the balance between its synthesis by the ChAT enzyme and its breakdown catalyzed by acetylcholinesterase (AChE) enzymes. It is striking that some parasitic nematodes that colonize mucosal surfaces encode additional AChE isoforms, which are produced in specific secretory glands and secreted into the worm environment[19] to most likely avoid detrimental exposure to host ACh.

Here, we investigated the specific role of tuft cell-derived ACh in the context of type 2 immune responses. We confirmed that tuft cells are the only intestinal epithelial cells expressing the key *Chat* gene for ACh biosynthesis, and they actually synthetize ACh. Worm clearance was delayed in mice with *Chat*-deficient intestinal epithelial cells in spite of the establishment of a strong type 2 immune response. Mechanistically, we demonstrated *in vivo* tuft cell ACh release into the gut lumen; *ex vivo*, we demonstrated a direct effect of ACh on worms, resulting in reduced fecundity via a muscarinic AChR-dependent pathway, as well as a transiently reduced mastocytosis in mice with *Chat*-deficient epithelial cells.

## RESULTS

### The gene encoding the ChAT enzyme is specifically expressed by intestinal epithelial tuft cells

To confirm the specific potential of small intestinal tuft cells to biosynthesize the ACh neurotransmitter, we assessed the expression of the gene encoding the choline acetyltransferase (ChAT) enzyme that catalyzes biosynthesis of ACh in fluorescence-activated cell sorting (FACS)-enriched small intestinal EpCam+; Siglec-F+ tuft cells as compared to the EpCam+; Siglec-F− non-tuft epithelial cell fraction. Efficiency of the cell-sorting procedure was assessed by analyzing the expression of the tuft cell marker *Trpm5* mRNA in the tuft and non-tuft cell fractions. The *Trpm5* mRNA was detected in the tuft cell fraction, whereas it was below the threshold of detection in the fraction containing non-tuft cells (Figure 1A). Similarly, the *Chat* mRNA was detected in the tuft cell fraction but not in the fraction containing all non-tuft cell epithelial subsets (EpCam+; Siglec-F−) of the small intestinal epithelium, indicating specific expression of the *Chat* gene in tuft cells among intestinal epithelial cells *in vivo* (Figure 1B). Then, to assess whether *Chat* mRNA expression is a common property of small intestinal tuft cells or is limited to only a subset of these cells, in the context of an intact tissue, we coupled anti-doublecortin-like kinase 1 (Dclk1) immunofluorescence detection of the entire tuft cell population to *in situ* hybridization to visualize *Chat*-expressing cells in naive C57BL/6 mice. This revealed specificity of the *Chat* probe signal in the vast majority of Dclk1-expressing tuft cells (Figure 1C). We then asked whether *Chat* expression is also a common feature of the amplified tuft cell population in the context of a helminth parasite infection and an ongoing type 2 immune response.[1,3] Wild-type mice were thus infected by gavage with *Heligmosomoides polygyrus* infective L3 larvae, which, after reaching the small intestine, penetrate the submucosa. There, they undergo two developmental moults before emerging again around 10 days post-infection (dpi) as adult worms into the gut lumen, where they mate and produce eggs, which are passed out in the feces. The presence of *H. polygyrus* worms in the gut lumen was associated with a strongly polarized type 2 immune response,[20] which included amplification of tuft cells.[21] *Chat* mRNA expression was also restricted to tuft cells from *H. polygyrus*-infected mice (Figure 1D), indicating the absence of *de novo Chat* expression in non-tuft cells in the context of a type 2 immune response, and was detected in almost all tuft cells. Furthermore, a quantitative comparison revealed similar proportions of *Chat*$^+$ Dclk1-expressing tuft cells in naive (574/614 examined cells) and infected (435/500 examined cells) mice (Figure 1E). Thus, *Chat* mRNA expression was present specifically in tuft cells in the mouse small intestinal epithelium and could be detected in almost all tuft cells regardless of their infection status.

### Host defense against helminth parasites is impaired in ChAT-deficient mice

We then investigated the function of tuft cell-produced ACh in the context of an *in vivo* type 2 immune response. We generated an inducible deletion of the *Chat* gene specifically in the intestinal epithelium by crossing *Chat*$^{LoxP/LoxP}$[22] and *Villin-Cre*$^{ERT2}$ mice to express the Cre recombinase in intestinal epithelial cells in a tamoxifen-inducible manner.[23] To assess the efficiency of the *Villin-Cre*$^{ERT2}$-mediated recombination at the *Chat* gene locus, we amplified by PCR the sequence of the LoxP-flanked exon 8 of the *Chat* gene in tamoxifen-treated *Chat*$^{LoxP/LoxP}$ and *Chat*$^{LoxP/LoxP}$;*Villin-Cre*$^{ERT2}$ mice. All mice were treated daily with tamoxifen for 5 days and analyzed 5 weeks later (Figure 2A). *Chat* Exon 8 sequence was amplified with genomic DNA from *Chat*$^{LoxP/LoxP}$ mice-enriched epithelial cells but was undetectable in *Chat*$^{LoxP/LoxP}$;*Villin-Cre*$^{ERT2}$ mice. Simultaneous amplification of the *Villin-Cre*$^{ERT2}$ sequence confirmed the presence of the *Villin-Cre*$^{ERT2}$ transgene uniquely in *Chat*$^{LoxP/LoxP}$;*Villin-Cre*$^{ERT2}$ mice as compared to *Chat*$^{LoxP/LoxP}$ mice, as well as the integrity of the genomic DNA purified from *Chat*$^{LoxP/LoxP}$;*Villin-Cre*$^{ERT2}$ mice (Figure 2B). This indicated highly efficient recombination of the *Chat* locus by the *Villin-Cre*$^{ERT2}$ transgene. Moreover, since the *Villin* gene promoter is active in all intestinal epithelial cells, including stem cells,[23] Cre$^{ERT2}$ activation by tamoxifen causes permanent gene deletion in the intestinal epithelium of compound *Chat*$^{LoxP/LoxP}$;*Villin-Cre*$^{ERT2}$ mice. Indeed, exon 8 deletion was very stable in spite of the rapid

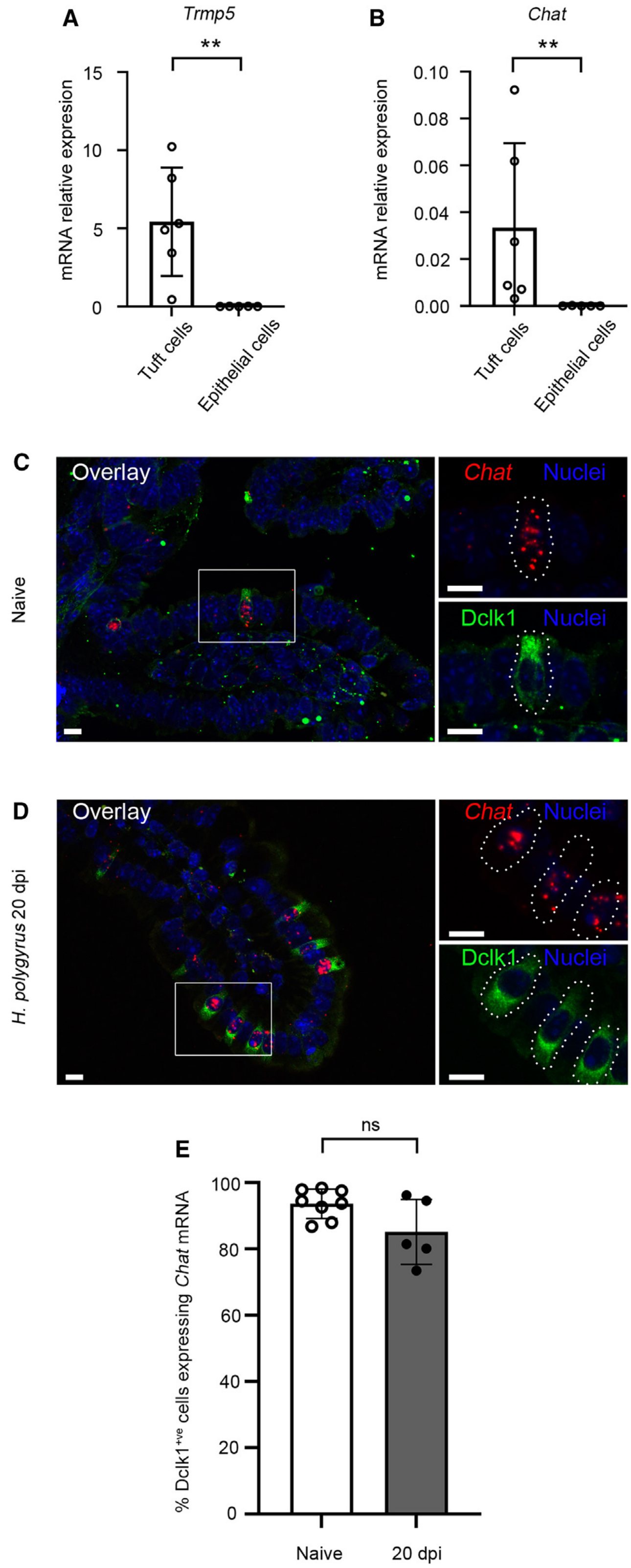


**Figure 1. *Chat* gene expression is restricted to tuft cells in the intestinal epithelium in naive mice and during *H. polygyrus* infection**

(A and B) Expression of the *Trpm5* (A) and *Chat* (B) transcripts in FACS-sorted tuft cells from C57BL/6J mice as compared to all other epithelial cells. Data represent median ± interquartile of the biological replicates ($n$ = 6 for tuft cell fractions, $n$ = 5 for all other epithelial cell fractions; Mann-Whitney test).

(C and D) Representative *in situ* hybridization for the *Chat* mRNA (red), coupled with immunofluorescence detection of Dclk1 (green) in naive (C) and *H. polygyrus*-infected (D) mice. Scale bars = 10 μm for all panels. Dotted lines show Dclk1-expressing tuft cells positive for the *Chat* transcript signal.

(E) Quantification of Dclk1 immunoreactive tuft cells expressing the *Chat* transcript in naive or *H. polygyrus*-infected mice. Data represent means ± SD of the biological replicates (based on quantification of 614 and 500 Dclk1-positive tuft cells, counted from $n$ = 8 and $n$ = 5 naive or infected mice, respectively; Student's t test).

renewal of the intestinal epithelial cells, as indicated by the absence of exon 8 sequence amplification from *Chat*$^{LoxP/LoxP}$;*Villin-Cre*$^{ERT2}$ mice intestinal epithelial cell genomic DNA five weeks after tamoxifen treatment (Figures 2A and 2B). Of note, *Villin-1* and *Chat* expression was reported in subsets of small intestinal type 2 innate lymphoid cell (ILC2) and ILC3 immune cell populations (Immunological Genome Project [ImmGen] database). To exclude any confounding non-epithelial Cre$^{ERT2}$ contribution in the analysis of the *Chat*$^{LoxP/LoxP}$;*Villin-Cre*$^{ERT2}$ mice, we investigated the expression of the *Villin-Cre*$^{ERT2}$ transgene product—more precisely, its nuclear translocation upon tamoxifen treatment—in small intestinal *lamina propria* ILC1, ILC2, and ILC3 subsets. Following tamoxifen injection (Figure 2C), nuclear Cre$^{ERT2}$ could be detected in all epithelial cells but neither in CD45$^{+}$ immune cells, nor, more specifically, in Tbx21+, Gata3+, and Rorγt+ immune subsets (Figure 2D), confirming the absence of *Villin-Cre*$^{ERT2}$ transgene expression in mouse ILC1, ILC2, and ILC3 subsets.

We then investigated the consequences of the epithelial *Chat* deficiency on the cellular composition of the gut mucosa. No gross alteration of the epithelial layer was perceptible following tamoxifen treatment of *Chat*$^{LoxP/LoxP}$;*Villin-Cre*$^{ERT2}$ mice as compared to *Chat*$^{LoxP/LoxP}$ control mice, as identical representations of epithelial cells from the goblet, Paneth, and enteroendocrine cell lineages were found in both mouse genotypes (Figure S1A). Moreover, the overall distribution of the immune subsets (T cells, B cells, and myeloid cell populations including monocytes, neutrophils, macrophages, and mast cells) within the hematopoietic cell compartment in the intestine (intraepithelial and *lamina propria* fractions), as well as in more distant peripheral lymphoid organs (spleen, mesenteric, and axillary/brachial/cervical lymph nodes), was equivalent between *Chat*$^{LoxP/LoxP}$;*Villin-Cre*$^{ERT2}$ and *Chat*$^{LoxP/LoxP}$ mice (Figures S1B and S1C). More specifically, no difference was observed in the CD4 and CD8 T cell subpopulation as well as in their activation and polarization status (Figure S1B). Similarly, the relative percentage of ILC1, ILC2, and ILC3 subsets was comparable in the *lamina propria* and intraepithelial populations of *Chat*$^{LoxP/LoxP}$;*Villin-Cre*$^{ERT2}$ and *Chat*$^{LoxP/LoxP}$ mice (Figure S1C). We then assessed the consequences of the ChAT deficiency during an infection with parasitic helminths. We first induced *Chat* gene deletion by tamoxifen treatment during five consecutive days. After five days of rest without tamoxifen, *Chat*$^{LoxP/LoxP}$ and *Chat*$^{LoxP/LoxP}$;*Villin-Cre*$^{ERT2}$ mice were infected by gavage with *H. polygyrus* L3, and infection parameters were analyzed from 10 to 40 days post-infection when adult worms are present in the gut lumen (Figure 3A). Although ChAT-deficient mice had

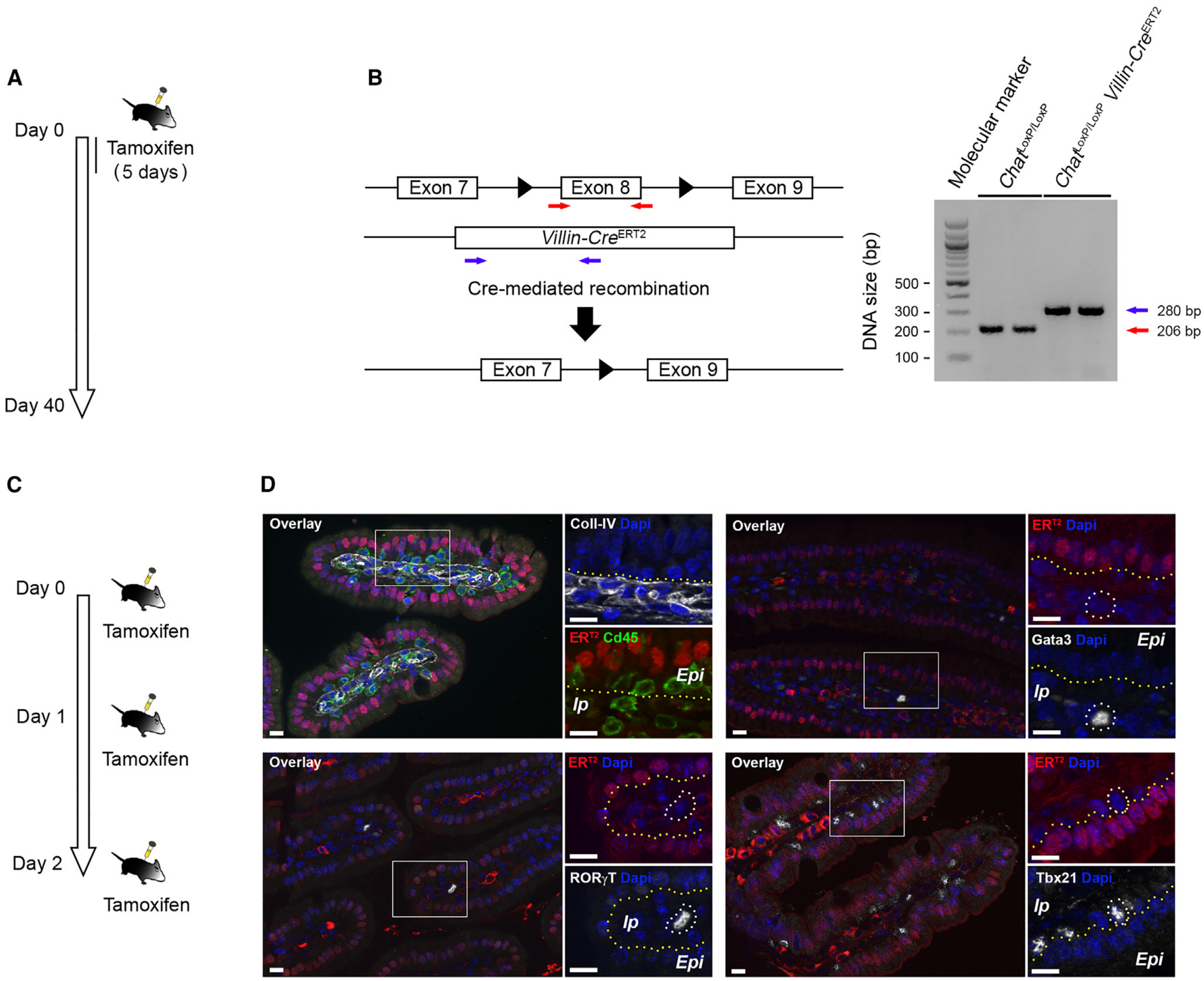


**Figure 2. Permanent and intestinal epithelial cell-restricted recombination driven by the *Villin-Cre*$^{ERT2}$ transgene**

(A) Mice from either *Chat*$^{LoxP/LoxP}$ or *Chat*$^{LoxP/LoxP}$;*Villin-Cre*$^{ERT2}$ genotypes were treated with tamoxifen during 5 consecutive days, and epithelial samples were recovered 5 weeks later.

(B) Agarose gel showing PCR reactions using primers specific to *Chat* exon 8 (red arrows in the scheme and electrophoresis; *n* = 2), as well as primers specific for the *Villin-Cre*$^{ERT2}$ transgene (blue arrows in the scheme and electrophoresis; *n* = 2). Upper bands (blue arrow) show specific signal related to presence of the *Villin-Cre*$^{ERT2}$ mice transgene.

(C) Mice were treated with tamoxifen for 2 consecutive days and immediately used for subsequent histological analyses.

(D) Immunohistochemistry (IHC) against the Cre$^{ERT2}$ fusion protein (red) co-stained with the pan-leukocyte lineages marker (CD45, green) in combination with collagen-IV, Tbx21, Gata3, and RORγt (gray). 136 RORγt-, 187 Gata3-, and 508 Tbx21-expressing cells were analyzed from *n* = 3 mice. Scale bars: 10μm. See also Figure S1.

no obvious phenotype, autopsy revealed increased numbers of adult worms in these mice as compared to *Chat*$^{LoxP/LoxP}$ control mice (Figure 3B). Greater numbers of live worms were mirrored by the numbers of eggs in the feces of infected mice, a dynamic readout of the type 2 immune response efficiency and worm persistence. Indeed, a significantly increased number of eggs was found in ChAT-deficient mice, as compared to *Chat*$^{LoxP/LoxP}$ littermates, between 10 days post-infection (when adult worms start to be present in the gut lumen) and 40 days post-infection (Figure 3C). Increased numbers of adult worms and fecal eggs in ChAT-deficient mice thus revealed an essential role of tuft cell-derived ACh for an efficient defense against helminth parasites.

## ACh concentrations are increased in tuft cells during type 2 immune responses

We then assessed the actual ACh concentration in tuft cells by mass spectrometry using lysates of FACS-sorted epithelial tuft cell fractions. ACh concentrations were significantly more elevated in the EpCam+; Siglec-F+ tuft cell fractions of mice infected with *H. polygyrus* as compared to the tuft cell fractions from naive mice, although high variability of ACh concentrations

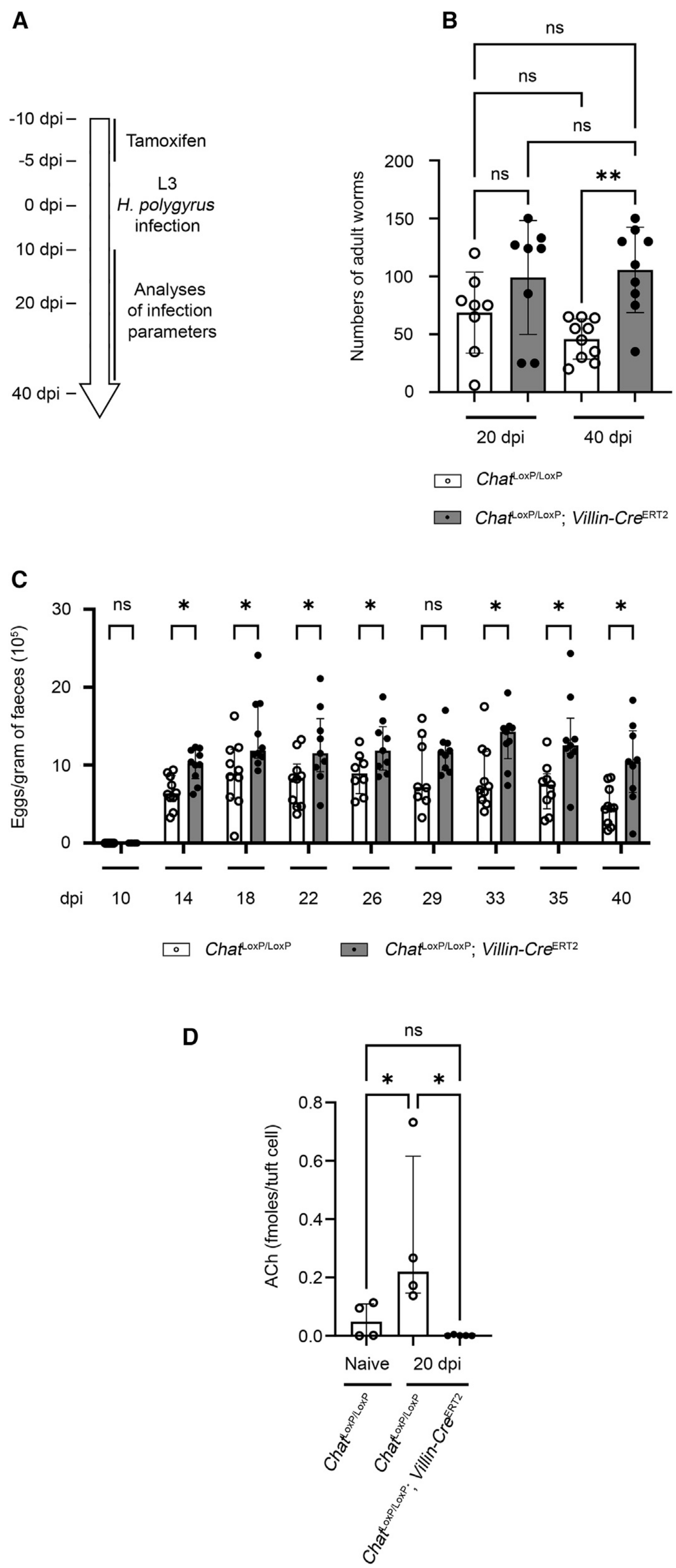


**Figure 3. Tuft cell *Chat* deficiency delays worm expulsion**

(A) Experimental design of worm infections and analysis time points. Mice were first treated with 1 mg of tamoxifen between −10 and −5 dpi and then orally infected with 200 *H. polygyrus* L3 larvae at day 0. Infected animals were monitored and/or sacrificed from 10 to 40 days post-infection.

(B) Numbers of adult worms found in infected $Chat^{LoxP/LoxP}$ (white bars and circles) or $Chat^{LoxP/LoxP}$;$Villin\text{-}Cre^{ERT2}$ mice (dark bars and circles). Data represent mean ± SD of the biological replicates ($n$ = 8 to 10 mice per time point; one-way ANOVA test).

(C) Numbers of eggs per gram of feces in infected $Chat^{LoxP/LoxP}$ (white bars and circles) or $Chat^{LoxP/LoxP}$;$Villin\text{-}Cre^{ERT2}$ mice (dark bars and circles). Data represent median ± interquartile of the biological replicates ($n$ = 8 to 10 mice per time point; multiple Mann-Whitney tests for each time point).

(D) ACh concentrations (fmoles per tuft cell) in tuft cells FACS-sorted from naive or infected $Chat^{LoxP/LoxP}$ and $Chat^{LoxP/LoxP}$;$Villin\text{-}Cre^{ERT2}$ mice. Bars represent medians ± interquartile of different biological samples ($n$ = 4 to 5 mice; Kruskal-Wallis analysis).

CellPress
OPEN ACCESS

was present in infected mice, likely reflecting difference in the infection efficiency (Figure 3D). Because this elevation occurred in cellular populations enriched in tuft cells, it likely reflected increased ACh synthesis per tuft cell in efficiently infected mice rather than a consequence of increased tuft cell numbers caused by the epithelial remodeling consequent to type 2 immune responses. As expected, ACh was not detected in the tuft cell fractions from *Chat*-deficient epithelial cells in *Chat*$^{LoxP/LoxP}$;*Villin-Cre*$^{ERT2}$ mice (Figure 3D). Thus, the basal tuft cell ACh biosynthesis rate significantly increased in the context of a type 2 immune response against *H. polygyrus* infection.

## ChAT-deficient mice are able to mount a type 2 immune response

To understand the mechanisms leading to decreased parasite clearance in *Chat*$^{LoxP/LoxP}$;*Villin-Cre*$^{ERT2}$ mice, we assessed critical parameters of the type 2 immune response and subsequent epithelial remodeling in naive and *H. polygyrus*-infected mice. Quantification of type 2 immune responses such as numbers of Gata3+ ILC2s/T helper 2 (Th2) cells, epithelial tuft cells, and goblet cells, and expression of the Retnlβ peptide by small intestinal goblet cells indicated the presence of a strong type 2 immune response in both *Chat*$^{LoxP/LoxP}$ control mice and *Chat*$^{LoxP/LoxP}$;*Villin-Cre*$^{ERT2}$ mice, with some of these parameters being even higher in infected ACh-deficient mice, possibly due to the presence of higher numbers of worms (Figures 4A–4C). To determine whether increased type 2 immunity parameters in infected ChAT-deficient mice were more likely caused by higher worm burden or directly consequent to the ChAT deficiency, we triggered a worm-independent activation of tuft cells and subsequent type 2 immune response by treating *Chat*$^{LoxP/LoxP}$ and *Chat*$^{LoxP/LoxP}$;*Villin-Cre*$^{ERT2}$ mice with succinate. Succinate is a known activator of tuft cells, the only intestinal epithelial cells expressing the succinate receptor 1 (Sucnr1) and subsequent type 2 immune responses.[24,25] Nearly identical type 2 immune responses, as assessed by quantification of *lamina propria* Gata3+ cells, epithelial tuft cells, and Retnlβ expression in goblet cells were observed in both mouse groups (Figure S2). This suggested that the increased type 2 immunity parameters in *H. polygyrus*-infected *Chat*$^{LoxP/LoxP}$;*Villin-Cre*$^{ERT2}$ mice were more likely caused by an increased worm burden than a direct effect of the ChAT deficiency. We also assessed the mRNA expression of tuft cell mediators of type 2 immune responses and factors known to be involved in ACh synthesis and transport, including the *Alox5*, *Alox5ap*, *Ltc4s*, *Ptgs1*, *Ptgs2*, *Hpgds*, *Pou2f3*, *Dclk1*, *Sucnr1*, *Il25*, *Chat*, and *VAChT* genes using intestinal epithelial cell extracts isolated from *Chat*$^{LoxP/LoxP}$ and *Chat*$^{LoxP/LoxP}$;*Villin-Cre*$^{ERT2}$ in naive and 20 and 40 dpi mice infected with *H. polygyrus*. As expected, the *Chat* mRNA was absent in extracts from *Chat*$^{LoxP/LoxP}$;*Villin-Cre*$^{ERT2}$ mice. With the exception of the *Dclk1* mRNA, which was found elevated in *Chat*$^{LoxP/LoxP}$;*Villin-Cre*$^{ERT2}$ mice at 40 days post-infection, no significant difference was found between control and ChAT-deficient mice at the naive or infected states (Figure S3). This was then confirmed at the cellular scale on tissue sections using immunofluorescence or *in situ* hybridization for all markers for which antibodies or probes were available (Alox5ap, Alox5, *Ltc4s*, Ptgs2, Hpgds, and *Sucnr1*). Again, no alteration in the expression patterns of these markers could be detected, suggesting that the ChAT deficiency did not alter substantially the expression of tuft cell alarmin molecules or their biosynthesis pathways (Figure S4), which is consistent with the observation of a strong type 2 immune response occurring in infected ChAT-deficient *Chat*$^{LoxP/LoxP}$;*Villin-Cre*$^{ERT2}$ mice.

To complement these data, we also sought to assess the distribution of the main immune cell populations present in the gut mucosa and distant lymphoid organs in control and ChAT-deficient infected mice. Unfortunately, the low viability of the cells dissociated from the inflamed gut mucosa of infected mice precluded such analyses. However, immunophenotyping could be performed on distant peripheral lymphoid organs (mesenteric lymph nodes, axillary/brachial/cervical lymph nodes, and spleen), and no significant differences were detected in ChAT-deficient infected mice as compared to controls (Figure S5 and STAR Methods). This suggested that the effects of tuft cell ACh deficiency remained local and did not affect distant or local lymphoid structures.

Mast cells constitute another immune subset involved in the defense against helminth parasites,[26] which can be analyzed by immunohistochemistry on inflamed tissue sections to circumvent the difficulty of studying dissociated cells from infected mice. We quantified the presence of mast cells in naive and infected mice using the mast cell protease 1 (Mcpt1) marker. In *Chat*$^{LoxP/LoxP}$;*Villin-Cre*$^{ERT2}$ mice, the Mcpt1+ cell population was more heterogeneous as compared to controls. Therefore, to quantify the difference in mast cell populations in control versus ChAT-deficient mice, we characterized the mast cell population detected in infected mice both in terms of percentage of the gut tissue with high density of mast cells and numbers of mast cells per microscopic field in regions of high mast cell densities (Videos S1, S2, S3, S4, S5, and S6). The rare Mcpt1+ cells found in naive *Chat*$^{LoxP/LoxP}$ mice strongly increased in *H. polygyrus*-infected mice 20 or 40 days post-infection. In ChAT-deficient mice, Mcpt1+ cell numbers also increased 20 days post infection but to a lesser extent compared to control mice, and at 40 days post infection, both *Chat*$^{LoxP/LoxP}$ and *Chat*$^{LoxP/LoxP}$;*Villin-Cre*$^{ERT2}$ mice had similarly elevated Mcpt1+ mast cell counts (Figures 5A–5C). In both genotypes, Mcpt1+ mast cells almost always co-expressed other mast cell markers such as CD117 (mostly referred to as cKit and Granzyme B [GzmB]), as well as CD63, suggesting an activated state (Figures 5D and S6A). Moreover, quantification of Mcpt1+ cells co-expressing CD63 revealed similar rates of mast cell activation between *Chat*$^{LoxP/LoxP}$ and *Chat*$^{LoxP/LoxP}$;*Villin-Cre*$^{ERT2}$ mice, with 92.9% and 97.3% double-positive cells at 20 dpi and 91.2% and 92.4% at 40 dpi, respectively (Figure S6B). Together, these data indicated that *Chat*$^{LoxP/LoxP}$;*Villin-Cre*$^{ERT2}$ ACh-deficient mice were able to mount a strong type 2 immune response in the absence of tuft cell-derived ACh, with the exception of mast cells, which were transiently less numerous at 20 days post-infection in ChAT-deficient mice. Thus, higher worm persistence and egg production in ChAT-deficient mice were not due to a globally compromised type 2 immune response, indicating a yet unappreciated role of ACh in promoting worm expulsion.

## Luminal ACh concentrations are increased following *H. polygyrus* infection

Certain helminth parasites express secreted isoforms of the AChE enzyme,[27] suggesting that degrading neighboring ACh is

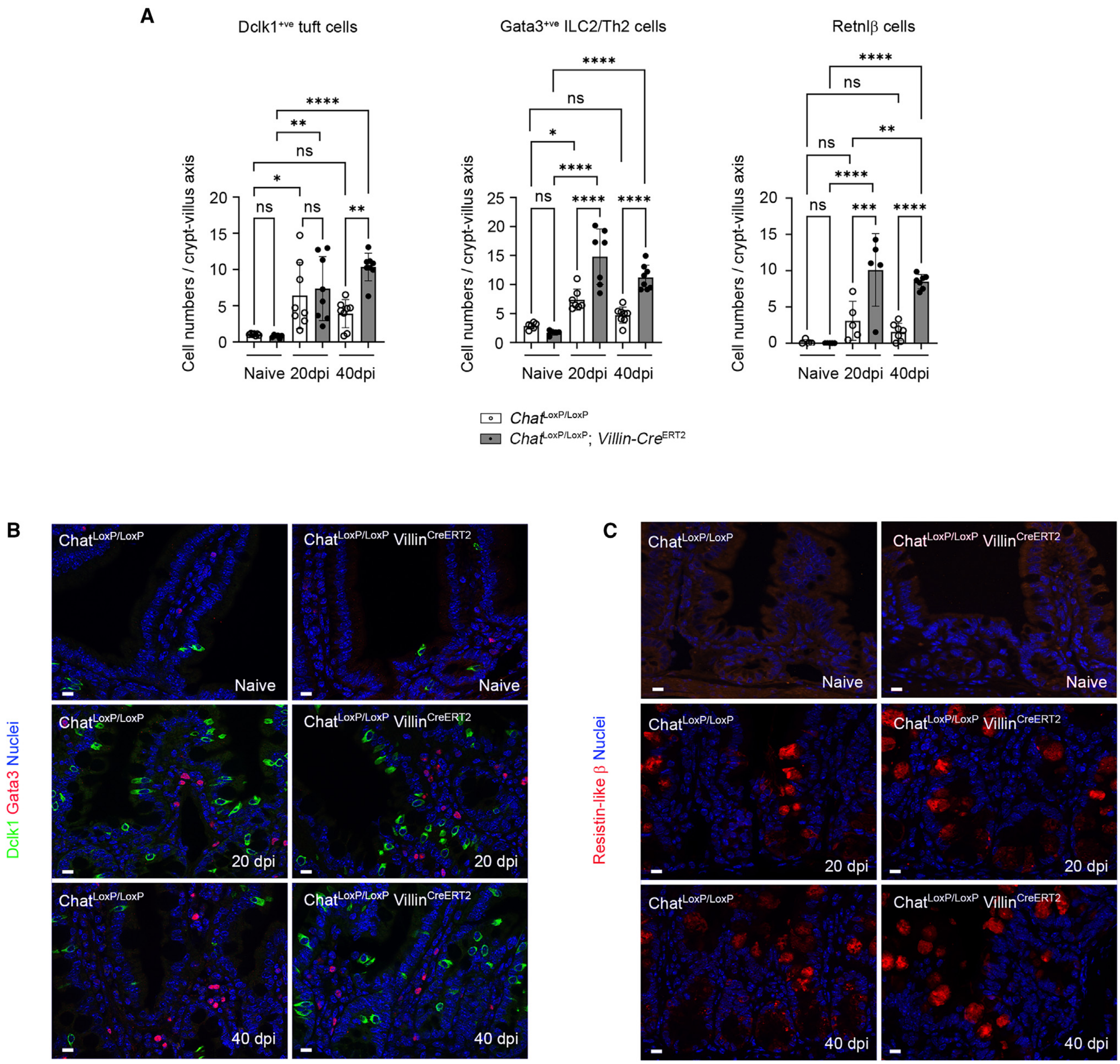


**Figure 4. *Chat* gene deficiency does not impair establishment of a type 2 immune response**

(A) IHC quantification using markers for tuft cells (Dclk1), ILC2s and Th2 immune cells (Gata3), and activated mucus-secreting goblet cells (resistin-like β) on intestinal sections of *Chat*$^{LoxP/LoxP}$ (white bars and circles) and *Chat*$^{LoxP/LoxP}$*;VillinCre*$^{ERT2}$ mice (dark bars and circles), naive or infected during the indicated time points. Bars represent means ± SD of the biological replicates ($n$ = 5 to 8; ANOVA analysis).

(B) Representative co-immunostainings of Dclk1 (green), Gata3 (red), and nuclei (blue) in naive or infected *Chat*$^{LoxP/LoxP}$ and *Chat*$^{LoxP/LoxP}$*;VillinCre*$^{ERT2}$ mice 20 and 40 days post infection.

(C) Representative co-immunostainings of Resistin-like β (red) and nuclei (blue) in naive or infected *Chat*$^{LoxP/LoxP}$ and *Chat*$^{LoxP/LoxP}$*;VillinCre*$^{ERT2}$ mice 20 and 40 days post infection. Scale bars = 10 μm for (B) and (C). See also Figures S2–S5.

beneficial for the worms. In order to investigate the hypothesis of a direct effect of tuft cell-derived ACh on worms present in the host gut lumen, we next assessed luminal ACh concentrations in naive and *H. polygyrus*-infected mice. For this, an intestinal loop was surgically ligatured and filled with wash buffer, which was recovered after 30 min of incubation and processed for detection of ACh by mass spectrometry. The resulting measurements revealed significantly more elevated ACh concentration in the gut lumen of control-infected mice compared to naive animals (Figure 6A), which was likely due to contribution of both higher tuft cell numbers and increased ACh production per tuft cell, as indicated by ACh quantification from extracts of FACS-sorted tuft cells as compared to identical numbers of non-tuft intestinal epithelial cells (Figure 3D). We noted that ACh

CellPress
OPEN ACCESS

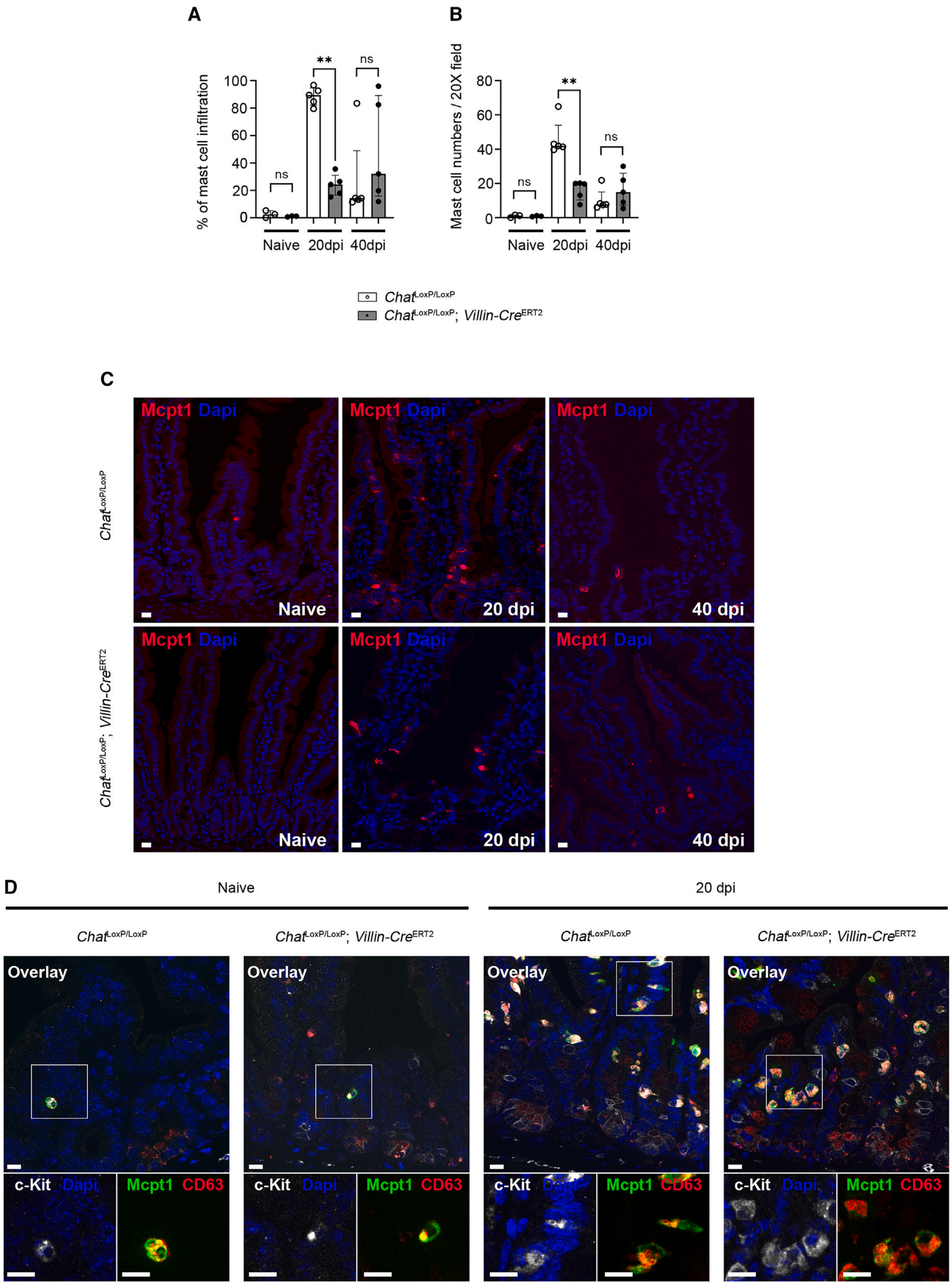


(legend on next page)

concentrations were variable in infected mice, probably reflecting infection efficiencies as well as the specific location of worms as regards the favorable intestinal loop for surgery. We then asked whether the actual presence of parasites was required for increased luminal ACh concentrations or whether this augmentation was directly linked to tuft cell activation. We thus treated mice with succinate and assessed ACh concentration changes. Indeed, luminal ACh was also significantly increased in the gut lumen of succinate-treated wild-type mice as compared to untreated mice, indicating luminal ACh release by tuft cells activated in the absence of worms (Figure 6B). Together, these data demonstrate the presence of tuft cell-derived ACh in the gut lumen of *H. polygyrus*-infected mice and suggest the possibility of a direct impact of luminal ACh on worm physiology.

### Increased worm fitness in mice with ACh-deficient tuft cells

To directly assess the consequences of *H. polygyrus* exposure to ACh, we used two complementary approaches. Noteworthily, intestinal goblet cells produce the Retnlβ molecule, specifically in the context of type 2 immune responses, and this directly interferes with *H. polygyrus* physiology.[4] Subsequently, it was found that worms exposed to Retnlβ had decreased ATP concentrations, reflecting altered viability and fecundity as compared to unexposed worms.[5,6] Thus, using a similar approach, we quantified ATP concentrations in worms recovered from the intestines of $Chat^{LoxP/LoxP}$ and $Chat^{LoxP/LoxP}$*;* *Villin-Cre*$^{ERT2}$ mice as a proxy of their global fitness. No significant difference was found in ATP concentrations of worms from mice of either genotype recovered at 20 days post-infection. In contrast, by day 40, significantly increased ATP concentrations were found in worms from mice with ACh-deficient tuft cells as compared to control $Chat^{LoxP/LoxP}$ littermates, suggesting decreased fitness of the worms obtained from an environment containing ACh for several weeks (Figure 6C).

### ACh exposure directly decreases worm fecundity

To confirm this finding, we also assessed worm fecundity *ex vivo* in the presence of 10 μM ACh, which is consistent with the micromolar range ACh concentrations found in the intestinal lumen lavage fluid obtained from *H. polygyrus*-infected $Chat^{LoxP/LoxP}$ control mice. Of note, it should also be taken into account the certainly partial recovery of luminal ACh using a single 30 min lavage as well as an estimated 15 times dilution factor corresponding to the lavage fluid volume injected into the intestinal loop (Figure S7). We thus quantified egg production per *H. polygyrus* adult female worm, freshly recovered from infected $Chat^{LoxP/LoxP}$ mice 14 days post-infection to ensure that the worms are present in the gut lumen and not yet strongly altered by luminal ACh. When ACh was added to the culture medium, egg production was significantly decreased as compared to untreated worms (Figure 6D). These data indicate that worm exposure to physiologically relevant ACh concentrations directly decrease helminth fecundity.

### ACh targets worm physiology via their muscarinic ACh receptors

To identify the mechanisms underlying the effect of ACh on worm physiology, we assessed worm fecundity[5,6] in the presence of ACh combined with different inhibitors of cholinergic signaling. As shown previously in Figure 6D, exposure to ACh alone decreased worm fecundity as compared to untreated worms. Exposure to ACh combined with mecamylamine, an antagonist of the nicotinic AChRs, caused a comparable significant drop in worm fecundity. In contrast, the inhibitory effect of ACh on egg production was not observed with worms treated with a combination of ACh and atropine, an antagonist of the muscarinic AChRs (Figure 6E). This implies that the inhibitory effects of ACh on worm fecundity were mediated by muscarinic AChRs expressed by the worms. Finally, none of the drugs had significant effects on egg production when used alone (Figure 6E). Altogether, these data argue that in addition to their sentinel function in initiating type 2 immune responses, intestinal tuft cells act as effectors of such responses by releasing into the host lumen non-neuronal ACh in a range of concentrations capable of directly altering helminth fecundity through their muscarinic AChRs as assessed in mechanistic *ex vivo* experiments (Figure 7). In addition, luminally or basolaterally secreted ACh likely contributes to the increased presence of activated mast cells following *H. polygyrus* infection.

## DISCUSSION

Thus far, the importance of tuft cell-produced ACh has been mostly studied in the airways. In the mouse tracheal epithelium, tuft cells are capable of sensing bitter compounds present in the airway lining fluid and use cholinergic signaling to neighboring nerve endings to cause an aversive reflex consisting of a reduced breathing frequency.[10] Tuft cells also participate in the regulation of bacterial populations present in the airway. These cells can be activated by bacterial quorum sensing molecules (QSMs)—used by microbes to evaluate their own population density—such as 3-Oxo$C_{12}$-HLS and use ACh signaling to cause respiratory changes.[16] In addition to respiratory reflexes, mucociliary clearance is an important innate protective process to eliminate inhaled pathogens from the airway. Paracrine cholinergic signaling from tracheal tuft cells—activated by bitter compounds, QSMs, or

**Figure 5. Tuft cell-restricted *Chat* gene deficiency impairs intestinal mastocytosis establishment following *H. polygyrus* infection**

(A) Intestinal mast cell infiltration score during *H. polygyrus* infection at the indicated time points, based on numbers of crypt-villus axes displaying two or more mast cell protease 1-expressing mast cells relative to the total number of crypt-villus axes of the small intestinal section. Data represent median ± interquartile of the biological replicates, ranging from $n = 3$ (naive), to $n = 5$ (for infected control and ChAT-deficient mice at 20 and 40 dpi); multiple Mann-Whitney test.

(B) Quantification of mast cell protease 1-expressing mast cells within small intestinal infiltrated areas. Data represent median ± interquartile of the biological replicates, ranging from $n = 3$ (naive) to $n = 5$ (for infected control and ChAT-deficient mice at 20 and 40 dpi, respectively); multiple Mann-Whitney test.

(C) Representative immunostainings of mast cell protease 1 (red) and nuclei (blue). Infection status, time points, and genotypes are indicated.

(D) Representative immunostainings of c-Kit (gray), Mcpt1 (green), and mast cell activation marker CD63 (red) and nuclei (blue). Infection status, time points, and genotypes are indicated. Scale bars = 10 μm for C and D. See also Figure S6 and Videos S1, S2, S3, S4, S5, and S6.

CellPress
OPEN ACCESS

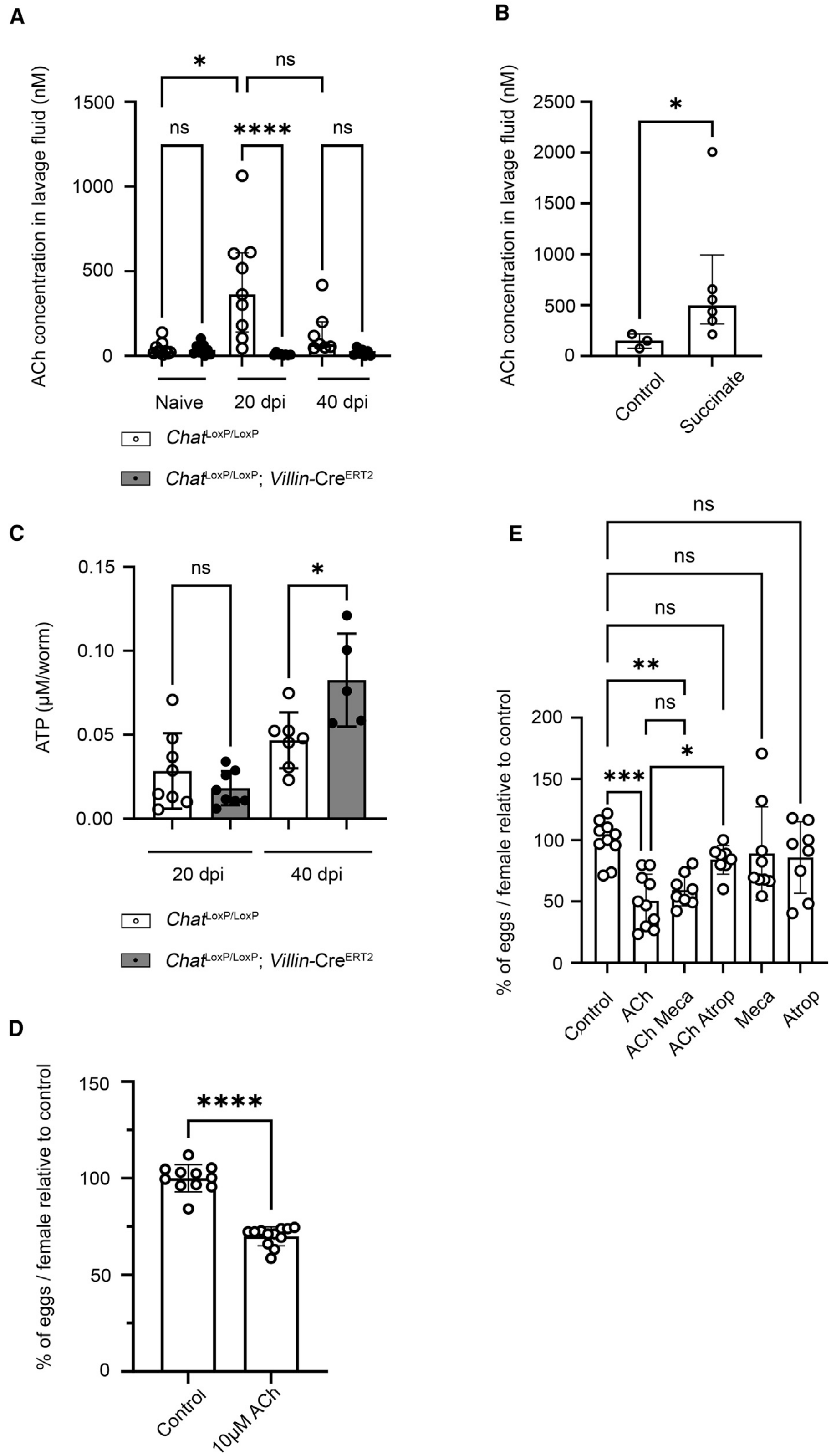


**Figure 6. Tuft cell-derived ACh is released into the host gut lumen, and ACh directly impairs worm physiology**

(A) Quantification of intestinal lavage fluid ACh concentration, as described in the STAR Methods section. Infection status, time points, and genotypes are indicated. Data represent median ± interquartile of the biological replicates ($n$ = 7 to 10); Kruskal-Wallis analysis.

*(legend continued on next page)*

other bacteria-derived products—was found to increase mucociliary clearance by airway ciliated cells, as assessed by the transport speed of particles on the tracheal surface, thereby directly linking chemosensation of bacterial signals with innate defense.[14,17] Previous studies with reporter mice also reported expression of *Chat*[11] and of the Tas2r143 bitter taste receptor[29] in a subset of tuft cells from the urogenital tract as well as from the gastrointestinal tract.[12] Furthermore, intraurethral application of a bitter compound regulated bladder activity in rats.[11] We confirmed intestinal tuft cell-restricted expression of the *Chat* gene for biosynthesis of ACh. Previous studies using green fluorescent protein (GFP) reporter driven by the *Chat* locus reported heterogeneity in GFP expression in trachea and urethra villin-immunoreactive tuft cells.[10,11] Instead, we found co-expression of the endogenous *Chat* mRNA and tuft cell Dclk1 protein in nearly all analyzed cells, suggesting either different regulation of *Chat* expression in the tuft cells from distinct organs or incomplete detection of the *Chat* gene product expression in genetically engineered reporter mice.

We present here two facets of a cholinergic function of small intestinal tuft cells. Firstly, we identified a regulator role on mastocytosis, the specific impact of which on the dynamics of *H. polygyrus* remains currently difficult to delineate. Secondly, we reported an effector function of tuft cells through the production of intestinal luminal ACh that may have direct effects on helminth parasites. Indeed, we reported the presence of ACh in the gut lumen with increased concentrations during type 2 immune responses in *H. polygyrus*-infected mice, reaching a micromolar range. Moreover, the increase in luminal ACh concentration could also be triggered by succinate treatment-driven tuft cell activation, suggesting that luminal ACh production is an integral component of the type 2 immune response downstream to tuft cell activation. Tuft cell-derived ACh directly or indirectly decreased worm viability 40 days post-infection, but not 20 days post-infection, as assessed by worm global ATP levels. Moreover, we demonstrated *ex vivo* a direct effect on worm fecundity following ACh exposure during 24 h, acting through *H. polygyrus* muscarinic AChRs. It is possible that *in vivo* quantification of global worm ATP concentrations, on the one hand, and *ex vivo* egg production, on the other hand, reflect ACh-dependent alterations of distinct physiological functions. Consistent with this, endogenous worm-derived ACh was reported to inhibit the egg laying behavior through worm muscarinic AChRs in the *Caenorhabditis elegans* helminth.[30] It is therefore plausible that host-derived ACh directly interferes with a related *H. polygyrus* muscarinic AChR pathway to decrease its fecundity. The reduced worm fitness observed 40 days post-infection might, in turn, rely on the alteration of distinct pathways, possibly involving nicotinic AChRs and related functions such as neuromuscular activity and feeding.[31]

Luminal ACh released by tuft cells in the context of a type 2 immune response illustrates how different epithelial subsets constitute integral parts of this process. Signaling through epithelial IL-4Rα receptors causes specific amplification of two epithelial cell lineages, tuft cells and goblet cells. In addition to the effector function of tuft cells discussed above, goblet cells not only contribute to the so-called weep and sweep response by increased mucus production, they also express *de novo* the Retnlβ peptide that directly interacts with the helminth's ability to feed on host tissues and thus also contribute to decreasing their fitness and fecundity.[4–6] As parasitic helminths have co-evolved with their hosts, it is plausible that the use of different defense mechanisms relying on distinct host cell types such as ACh and Retnlβ, produced by tuft and goblet cells, respectively, renders the task more challenging for parasites to counteract host immunity. In this view, it might be interesting to combine both ACh and Retnlβ host deficiencies to investigate possible synergistic action of these two molecules on different helminth parasite species.

Cholinergic regulation of immune responses is well described,[32] including in the context of helminth infections.[33–36] In the current study, we did not investigate the consequences of tuft cell ACh deficiency on immune subsets in more detail, with the exception of intestinal mucosal mast cells. This limited exploration was justified, because despite a strong type 2 immune response, no additional alterations in the expression of tuft cell-derived immune mediators, nor significant alterations of immune populations, were identified in *Chat*$^{LoxP/LoxP}$;*Villin-Cre*$^{ERT2}$ mice. We, however, reported that the mastocytosis observed following *H. polygyrus* infection was partially inhibited in mice with ChAT-deficient tuft cells. Although the precise links between mast cells and type 2 immune responses remain only partially understood, multiple studies reported decreased anti-parasitic defense in mouse lines with mast cell-deficiencies, including after infection with *H. polygyrus*.[37,38] Thus, although mastocytosis following helminth infection occurred in the absence of tuft cell-derived ACh, it was quantitatively lower as compared to infected control mice at 20 days post-infection, which might contribute to the delayed control of *H. polygyrus* infection observed in *Chat*$^{LoxP/LoxP}$;*Villin-Cre*$^{ERT2}$ mice. Noteworthily, several studies on the role of mast cells during helminth infections used *cKit* gene loss of function models[37,39,40] or its pharmacological inhibition[38,41] to cause mast cell deficiencies. cKit is also expressed in hematopoietic progenitors,[42] as well as in epithelial Paneth,[43] goblet,[44] and tuft cells,[45] which might be confounding if a tuft cell deficiency was induced beside the mast cell deficiency.

(B) Quantification of intestinal lavage fluid ACh concentration, as described in the STAR Methods section, in naïve or succinate-treated mice (one week treatment with 100 mM succinate in the drinking water). Bars represent median ± interquartile of the biological replicates ($n$ = 3 to 6); Mann-Whitney test. For (A) and (B), ACh concentrations were determined in the intestinal lavage fluid recovered per animal.

(C) ATP levels (μM) in worms. Infection time points and genotypes are indicated. Data represent mean ± SD of the different biological replicates ($n$ = 5 to 8 groups of 25 worms per time point; ANOVA analysis).

(D) Percentage of eggs released in the culture medium during a 24 h time frame after treatment of adult *H. polygyrus* worms with 10 μM ACh, as compared to untreated control worms. Bars represent mean ± SD of the biological replicates ($n$ = 11 to 12 groups of 25 female worms per condition; Student's t test).

(E) Percentage of eggs released in the culture medium during a 24 h time frame after treatment of adult *H. polygyrus* worms with 10 μM ACh (ACh, $n$ = 10); 10μM ACh combined with 5 μM mecamylamine (ACh Meca, $n$ = 8) or 5 μM atropine (ACh Atrop, $n$ = 8); or 5 μM mecamylamine or 5 μM atropine alone (Meca and Atrop, $n$ = 9 and 8, respectively), as compared to untreated control worms ($n$ = 10). Bars represent mean ± SD of the biological replicates; one-way ANOVA test. See also Figure S7.

CellPress
OPEN ACCESS

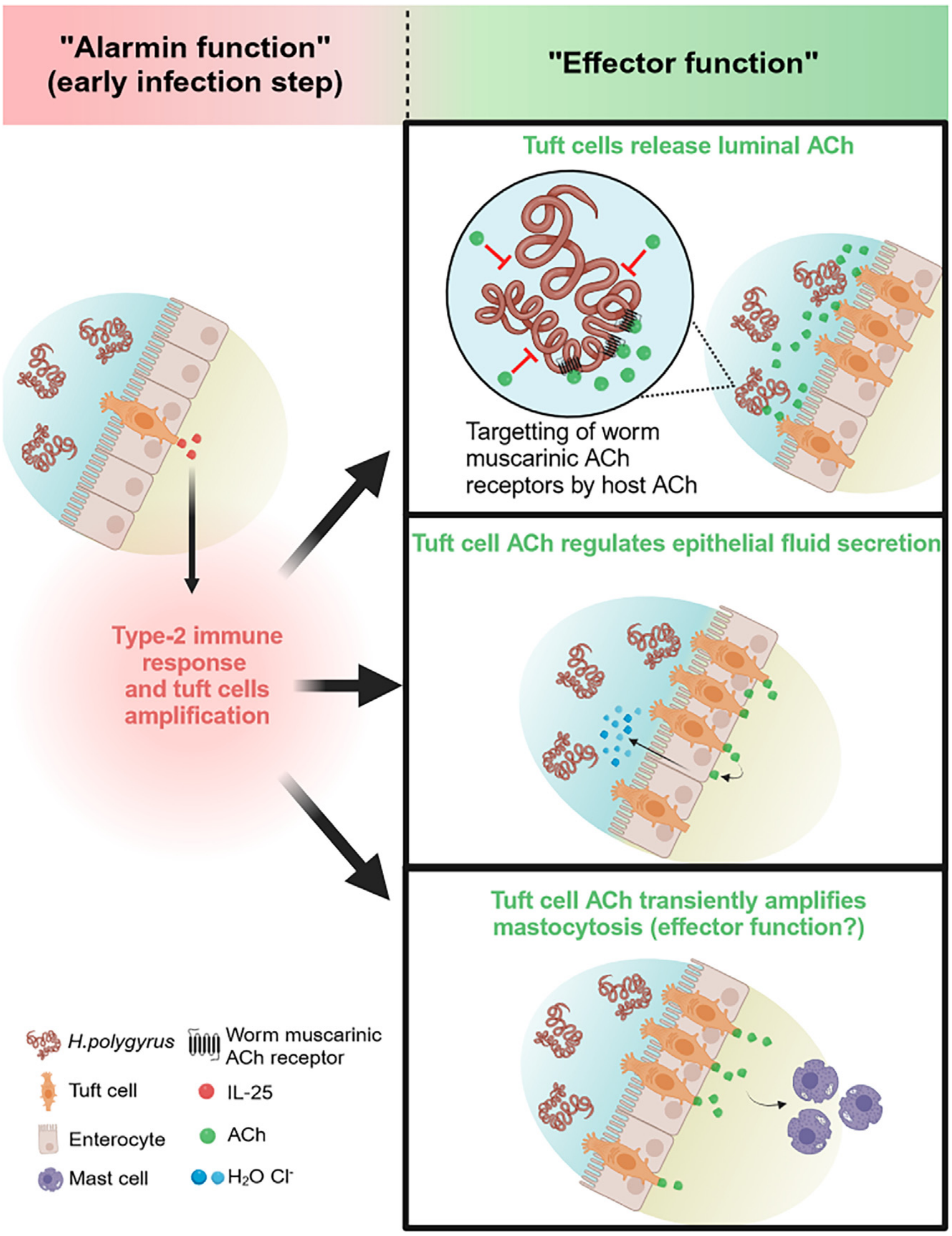


**Figure 7. Scheme illustrating the tuft cell effector function during helminth parasite infection**

After initial detection of helminths, tuft cells secrete alarmins to initiate a type 2 immune response (left), as previously described, eventually leading to mucosal remodeling and drastic tuft cell amplification. Newly produced tuft cells are able to release ACh (green dots) into the lumen to target helminths through their muscarinic receptors (upper right) and trigger fluid secretion from neighboring epithelial cells (middle right), as shown in Billipp et al.[28] Tuft cell-derived ACh also transiently enhances mastocytosis (lower right).

Luminal release of ACh and fine-tuning of mucosal mastocytosis may not be the sole modes of action of tuft cell ACh in the small intestine. Additional cholinergic mechanisms, such as paracrine signaling to other epithelial cells or nerve cells, as reported in the airway,[10,14] were recently reported in the small intestine.[28] Moreover, additional ACh sources, such as ILC2s, exist in the gut mucosa and also contribute to ILC2 numbers inflation and anti-helminth immunity.[35]

Our study on the cholinergic functions of intestinal tuft cells thus revealed additional functions for these cells with a luminal mode of action of ACh. Hence, we propose that tuft cells play two distinct but complementary roles in the context of the host defense against helminth infections. In the naive mucosa, tuft cells are a rare epithelial subset functioning as a sentinel to initiate type 2 immune responses upon parasitic infections. Our findings now document how the amplification of the tuft cell lineage, occurring in the context of an ongoing type 2 immune response, underlies a second function as a cholinergic regulator of mastocytosis and effector that likely directly contributes to parasitic helminth clearance by compromising their global fitness. Moreover, as demonstrated *ex vivo*, ACh concentrations measured in the gut lumen can directly alter *H. polygyrus* fecundity. This not only highlights the role of tuft cell hyperplasia during type 2 immune responses but may also provide a missing link to understand why IL-25 deficiency, a key tuft cell cytokine involved in their sentinel function,[8] causes a less severe phenotype than the complete absence of tuft cells in Pou2f3-deficient mice.[12]

## Limitations of the study

Despite these insights, several questions remain. First, the tuft cell mechanisms underlying ACh secretion remain unclear. A non-canonical pathway might be involved since previous expression studies on the VAChT protein, required for the uptake of ACh into small synaptic vesicles, and the high-affinity choline transporter (ChT1), required for the re-uptake of choline to promote intracellular ACh synthesis, reported the absence of detectable expression of these two proteins in tuft cells from all intestinal segments.[12] Second, our study introduces the notion that tuft cells release ACh as a molecule that directly targets helminth parasites. This notion might be expanded with the discovery of additional tuft cell factors contributing to the same function against helminths or other intestinal parasites. Finally, future studies will be needed to evaluate the potential therapeutic perspectives consecutive to our findings in refining the common anti-helminth treatments of humans or livestock, such as cholinergic agonists or inhibitors of AChEs, with a focus on a luminal mode of action of these drugs.

## STAR★METHODS

Detailed methods are provided in the online version of this paper and include the following:

- KEY RESOURCES TABLE
- RESOURCE AVAILABILITY
  - Lead contact

○ Materials availability
○ Data and code availability
● EXPERIMENTAL MODEL AND STUDY PARTICIPANT DETAILS
○ Mouse models
● METHOD DETAILS
○ Animal procedures
○ Collection of adults *H. polygyrus* and *in vitro* culture for treatment with cholinergic drugs
○ Cell sorting experiments
○ Immunophenotyping and flow cytometric analysis
○ ACh quantification
○ RNA extraction and PCR
○ Fluorescent immunohistochemistry or *in situ* hybridization on paraffin-embedded tissue
○ Microscopy and imaging
● QUANTIFICATION AND STATISTICAL ANALYSES

## SUPPLEMENTAL INFORMATION

Supplemental information can be found online at https://doi.org/10.1016/j.immuni.2024.04.018.

## ACKNOWLEDGMENTS

We acknowledge Prof. M. Selkirk for scientific discussions, Dr. E. Valjent for technical inputs and reagents, Elisa Evain for help in immunophenotyping experiments, the RAM-iExplore and RAM-PCEA facilities for maintenance of mouse colonies, and the Montpellier RIO Imaging and Arpege facilities. This work was supported by a Wellcome Trust Collaborative Award (ref. 211814) to P.J., C.B., E.D., T.M., and R.M.; Agence Nationale de la Recherche (ANR-17-CE15-0016-01 and ANR-21-CE15-0017-01 to P.J.); Institut National Du Cancer (INCA_2018-158 to P.J.)—the P.J. team is "Equipe Labellisée Ligue contre le Cancer"; M.N. was supported by the Labex EpiGenMed (an "Investissements d'avenir" program ANR-10-LABX-12-01) and the Wellcome Trust Collaborative Award (ref. 211814). We acknowledge the French Ministry of Higher Education and Research for supporting the PLATON facility through a CPER grant (IBDLR) as well as Oriane Scholler (PLATON) for technical discussion.

## AUTHOR CONTRIBUTIONS

Conceptualization, P.J., F.G., and R.M.M.; methodology, M.D., F.H., C.J., A.T., and S.T.; investigation, M.D., F.G., F.H., I.G., C.J., J.B., A.L., N.C., S.H., V.D., V.S.Z., M.C.P., E.T., A.G., and C.C.; writing—original draft, P.J. and F.G.; writing—review & editing, P.J., F.G., R.M.M., C.B., E.D., T.N.M., V.D., V.S.Z., A.T., and S.B.; funding acquisition, R.M.M., P.J., C.B., E.D., and T.N.M.; resources, S.B., Z.H., J.P., L.F., and C.C.; supervision, P.J., F.G., and R.M.M.

## DECLARATION OF INTERESTS

The authors declare no competing interests.

Received: June 18, 2023
Revised: November 26, 2023
Accepted: April 17, 2024
Published: June 11, 2024

## REFERENCES

1. Gerbe, F., Sidot, E., Smyth, D.J., Ohmoto, M., Matsumoto, I., Dardalhon, V., Cesses, P., Garnier, L., Pouzolles, M., Brulin, B., et al. (2016). Intestinal epithelial tuft cells initiate type 2 mucosal immunity to helminth parasites. Nature *529*, 226–230. https://doi.org/10.1038/nature16527.
2. Howitt, M.R., Lavoie, S., Michaud, M., Blum, A.M., Tran, S.V., Weinstock, J.V., Gallini, C.A., Redding, K., Margolskee, R.F., Osborne, L.C., et al. (2016). Tuft cells, taste-chemosensory cells, orchestrate parasite type 2 immunity in the gut. Science *351*, 1329–1333. https://doi.org/10.1126/science.aaf1648.
3. von Moltke, J., Ji, M., Liang, H.-E., and Locksley, R.M. (2016). Tuft-cell-derived IL-25 regulates an intestinal ILC2–epithelial response circuit. Nature *529*, 221–225. https://doi.org/10.1038/nature16161.
4. Artis, D., Wang, M.L., Keilbaugh, S.A., He, W., Brenes, M., Swain, G.P., Knight, P.A., Donaldson, D.D., Lazar, M.A., Miller, H.R.P., et al. (2004). RELMbeta/FIZZ2 is a goblet cell-specific immune-effector molecule in the gastrointestinal tract. Proc. Natl. Acad. Sci. USA *101*, 13596–13600. https://doi.org/10.1073/pnas.0404034101.
5. Herbert, D.R., Yang, J.Q., Hogan, S.P., Groschwitz, K., Khodoun, M., Munitz, A., Orekov, T., Perkins, C., Wang, Q., Brombacher, F., et al. (2009). Intestinal epithelial cell secretion of RELM-beta protects against gastrointestinal worm infection. J. Exp. Med. *206*, 2947–2957. https://doi.org/10.1084/jem.20091268.
6. Chen, G., Wang, S.H., Jang, J.C., Odegaard, J.I., and Nair, M.G. (2016). Comparison of RELMα and RELMβ Single- and Double-Gene-Deficient Mice Reveals that RELMα Expression Dictates Inflammation and Worm Expulsion in Hookworm Infection. Infect. Immun. *84*, 1100–1111. https://doi.org/10.1128/IAI.01479-15.
7. Molofsky, A.B., and Locksley, R.M. (2023). The ins and outs of innate and adaptive type 2 immunity. Immunity *56*, 704–722. https://doi.org/10.1016/j.immuni.2023.03.014.
8. Fallon, P.G., Ballantyne, S.J., Mangan, N.E., Barlow, J.L., Dasvarma, A., Hewett, D.R., McIlgorm, A., Jolin, H.E., and McKenzie, A.N.J. (2006). Identification of an interleukin (IL)-25-dependent cell population that provides IL-4, IL-5, and IL-13 at the onset of helminth expulsion. J. Exp. Med. *203*, 1105–1116. https://doi.org/10.1084/jem.20051615.
9. Wessler, I., and Kirkpatrick, C.J. (2020). Cholinergic signaling controls immune functions and promotes homeostasis. Int. Immunopharmacol. *83*, 106345. https://doi.org/10.1016/j.intimp.2020.106345.
10. Krasteva, G., Canning, B.J., Hartmann, P., Veres, T.Z., Papadakis, T., Mühlfeld, C., Schliecker, K., Tallini, Y.N., Braun, A., Hackstein, H., et al. (2011). Cholinergic chemosensory cells in the trachea regulate breathing. Proc. Natl. Acad. Sci. USA *108*, 9478–9483. https://doi.org/10.1073/pnas.1019418108.
11. Deckmann, K., Filipski, K., Krasteva-Christ, G., Fronius, M., Althaus, M., Rafiq, A., Papadakis, T., Renno, L., Jurastow, I., Wessels, L., et al. (2014). Bitter triggers acetylcholine release from polymodal urethral chemosensory cells and bladder reflexes. Proc. Natl. Acad. Sci. USA *111*, 8287–8292. https://doi.org/10.1073/pnas.1402436111.
12. Schütz, B., Jurastow, I., Bader, S., Ringer, C., von Engelhardt, J., Chubanov, V., Gudermann, T., Diener, M., Kummer, W., Krasteva-Christ, G., and Weihe, E. (2015). Chemical coding and chemosensory properties of cholinergic brush cells in the mouse gastrointestinal and biliary tract. Front. Physiol. *6*, 87. https://doi.org/10.3389/fphys.2015.00087.
13. Panneck, A.R., Rafiq, A., Schütz, B., Soultanova, A., Deckmann, K., Chubanov, V., Gudermann, T., Weihe, E., Krasteva-Christ, G., Grau, V., et al. (2014). Cholinergic epithelial cell with chemosensory traits in murine thymic medulla. Cell Tissue Res. *358*, 737–748. https://doi.org/10.1007/s00441-014-2002-x.
14. Perniss, A., Liu, S., Boonen, B., Keshavarz, M., Ruppert, A.-L., Timm, T., Pfeil, U., Soultanova, A., Kusumakshi, S., Delventhal, L., et al. (2020). Chemosensory Cell-Derived Acetylcholine Drives Tracheal Mucociliary Clearance in Response to Virulence-Associated Formyl Peptides. Immunity *52*, 683–699.e11. https://doi.org/10.1016/j.immuni.2020.03.005.
15. Saunders, C.J., Christensen, M., Finger, T.E., and Tizzano, M. (2014). Cholinergic neurotransmission links solitary chemosensory cells to nasal inflammation. Proc. Natl. Acad. Sci. USA *111*, 6075–6080. https://doi.org/10.1073/pnas.1402251111.
16. Krasteva, G., Canning, B.J., Papadakis, T., and Kummer, W. (2012). Cholinergic brush cells in the trachea mediate respiratory responses to quorum sensing molecules. Life Sci. *91*, 992–996. https://doi.org/10.1016/j.lfs.2012.06.014.
17. Hollenhorst, M.I., Jurastow, I., Nandigama, R., Appenzeller, S., Li, L., Vogel, J., Wiederhold, S., Althaus, M., Empting, M., Altmüller, J., et al. (2020). Tracheal brush cells release acetylcholine in response to bitter

CellPress
OPEN ACCESS

tastants for paracrine and autocrine signaling. FASEB J *34*, 316–332. https://doi.org/10.1096/fj.201901314RR.

18. Martin, R.J., and Robertson, A.P. (2007). Mode of action of levamisole and pyrantel, anthelmintic resistance, E153 and Q57. Parasitology *134*, 1093–1104. https://doi.org/10.1017/S0031182007000029.

19. Selkirk, M.E., Lazari, O., Hussein, A.S., and Matthews, J.B. (2005). Nematode acetylcholinesterases are encoded by multiple genes and perform non-overlapping functions. Chem. Biol. Interact. *157–158*, 263–268. https://doi.org/10.1016/j.cbi.2005.10.039.

20. Reynolds, L.A., Filbey, K.J., and Maizels, R.M. (2012). Immunity to the model intestinal helminth parasite Heligmosomoides polygyrus. Semin. Immunopathol. *34*, 829–846. https://doi.org/10.1007/s00281-012-0347-3.

21. Drurey, C., Lindholm, H.T., Coakley, G., Poveda, M.C., Löser, S., Doolan, R., Gerbe, F., Jay, P., Harris, N., Oudhoff, M.J., and Maizels, R.M. (2022). Intestinal epithelial tuft cell induction is negated by a murine helminth and its secreted products. J. Exp. Med. *219*, e20211140. https://doi.org/10.1084/jem.20211140.

22. Lecomte, M.-J., Bertolus, C., Santamaria, J., Bauchet, A.-L., Herbin, M., Saurini, F., Misawa, H., Maisonobe, T., Pradat, P.-F., Nosten-Bertrand, M., et al. (2014). Selective disruption of acetylcholine synthesis in subsets of motor neurons: a new model of late-onset motor neuron disease. Neurobiol. Dis. *65*, 102–111. https://doi.org/10.1016/j.nbd.2014.01.014.

23. El Marjou, F., Janssen, K.-P., Chang, B.H.J., Li, M., Hindie, V., Chan, L., Louvard, D., Chambon, P., Metzger, D., and Robine, S. (2004). Tissue-specific and inducible Cre-mediated recombination in the gut epithelium. genesis *39*, 186–193. https://doi.org/10.1002/gene.20042.

24. Nadjsombati, M.S., McGinty, J.W., Lyons-Cohen, M.R., Jaffe, J.B., DiPeso, L., Schneider, C., Miller, C.N., Pollack, J.L., Nagana Gowda, G.A., Fontana, M.F., et al. (2018). Detection of Succinate by Intestinal Tuft Cells Triggers a Type 2 Innate Immune Circuit. Immunity *49*, 33–41.e7. https://doi.org/10.1016/j.immuni.2018.06.016.

25. Schneider, C., O'Leary, C.E., von Moltke, J., Liang, H.-E., Ang, Q.Y., Turnbaugh, P.J., Radhakrishnan, S., Pellizzon, M., Ma, A., and Locksley, R.M. (2018). A Metabolite-Triggered Tuft Cell-ILC2 Circuit Drives Small Intestinal Remodeling. Cell *174*, 271–284.e14. https://doi.org/10.1016/j.cell.2018.05.014.

26. Inclan-Rico, J.M., and Siracusa, M.C. (2018). First responders: innate immunity to helminths. Trends Parasitol. *34*, 861–880. https://doi.org/10.1016/j.pt.2018.08.007.

27. Selkirk, M.E., Lazari, O., and Matthews, J.B. (2005). Functional genomics of nematode acetylcholinesterases. Parasitology *131*, S3–S18. https://doi.org/10.1017/S0031182005008206.

28. Billipp, T.E., Fung, C., Webeck, L.M., Sargent, D.B., Gologorsky, M.B., McDaniel, M.M., Kasal, D.N., McGinty, J.W., Barrow, K.A., Rich, L.M., et al. (2023). Tuft cell-derived acetylcholine regulates epithelial fluid secretion. Preprint at bioRxiv. https://doi.org/10.1101/2023.03.17.533208.

29. Liu, S., Lu, S., Xu, R., Atzberger, A., Günther, S., Wettschureck, N., and Offermanns, S. (2017). Members of Bitter Taste Receptor Cluster Tas2r143/Tas2r135/Tas2r126 Are Expressed in the Epithelium of Murine Airways and Other Non-gustatory Tissues. Front. Physiol. *8*, 849. https://doi.org/10.3389/fphys.2017.00849.

30. Bany, I.A., Dong, M.-Q., and Koelle, M.R. (2003). Genetic and Cellular Basis for Acetylcholine Inhibition of Caenorhabditis elegans Egg-Laying Behavior. J. Neurosci. *23*, 8060–8069. https://doi.org/10.1523/JNEUROSCI.23-22-08060.2003.

31. You, H., Liu, C., Du, X., and McManus, D.P. (2017). Acetylcholinesterase and Nicotinic Acetylcholine Receptors in Schistosomes and Other Parasitic Helminths. Molecules *22*, 1550. https://doi.org/10.3390/molecules22091550.

32. Rosas-Ballina, M., and Tracey, K.J. (2009). Cholinergic control of inflammation. J. Intern. Med. *265*, 663–679. https://doi.org/10.1111/j.1365-2796.2009.02098.x.

33. Darby, M., Schnoeller, C., Vira, A., Culley, F.J., Bobat, S., Logan, E., Kirstein, F., Wess, J., Cunningham, A.F., Brombacher, F., et al. (2015). The M3 Muscarinic Receptor Is Required for Optimal Adaptive Immunity to Helminth and Bacterial Infection. PLoS Pathog. *11*, e1004636. https://doi.org/10.1371/journal.ppat.1004636.

34. Vaux, R., Schnoeller, C., Berkachy, R., Roberts, L.B., Hagen, J., Gounaris, K., and Selkirk, M.E. (2016). Modulation of the Immune Response by Nematode Secreted Acetylcholinesterase Revealed by Heterologous Expression in Trypanosoma musculi. PLoS Pathog. *12*, e1005998. https://doi.org/10.1371/journal.ppat.1005998.

35. Chu, C., Parkhurst, C.N., Zhang, W., Zhou, L., Yano, H., Arifuzzaman, M., and Artis, D. (2021). The ChAT-acetylcholine pathway promotes group 2 innate lymphoid cell responses and anti-helminth immunity. Sci. Immunol. *6*, eabe3218. https://doi.org/10.1126/sciimmunol.abe3218.

36. Roberts, L.B., Schnoeller, C., Berkachy, R., Darby, M., Pillaye, J., Oudhoff, M.J., Parmar, N., Mackowiak, C., Sedda, D., Quesniaux, V., et al. (2021). Acetylcholine production by group 2 innate lymphoid cells promotes mucosal immunity to helminths. Sci. Immunol. *6*, eabd0359. https://doi.org/10.1126/sciimmunol.abd0359.

37. Hepworth, M.R., Daniłowicz-Luebert, E., Rausch, S., Metz, M., Klotz, C., Maurer, M., and Hartmann, S. (2012). Mast cells orchestrate type 2 immunity to helminths through regulation of tissue-derived cytokines. Proc. Natl. Acad. Sci. *109*, 6644–6649. https://doi.org/10.1073/pnas.1112268109.

38. Shimokawa, C., Kanaya, T., Hachisuka, M., Ishiwata, K., Hisaeda, H., Kurashima, Y., Kiyono, H., Yoshimoto, T., Kaisho, T., and Ohno, H. (2017). Mast Cells Are Crucial for Induction of Group 2 Innate Lymphoid Cells and Clearance of Helminth Infections. Immunity *46*, 863–874.e4. https://doi.org/10.1016/j.immuni.2017.04.017.

39. Alizadeh, H., and Murrell, K.D. (1984). The intestinal mast cell response to Trichinella spiralis infection in mast cell-deficient w/wv mice. J. Parasitol. *70*, 767–773.

40. González, M.I., Lopes, F., McKay, D.M., and Reyes, J.L. (2018). Mast cell deficiency in mice results in biomass overgrowth and delayed expulsion of the rat tapeworm Hymenolepis diminuta. Biosci. Rep. *38*, BSR20180687. https://doi.org/10.1042/BSR20180687.

41. Donaldson, L.E., Schmitt, E., Huntley, J.F., Newlands, G.F., and Grencis, R.K. (1996). A critical role for stem cell factor and c-kit in host protective immunity to an intestinal helminth. Int. Immunol. *8*, 559–567. https://doi.org/10.1093/intimm/8.4.559.

42. Tsai, M., Valent, P., and Galli, S.J. (2022). KIT as a master regulator of the mast cell lineage. J. Allergy Clin. Immunol. *149*, 1845–1854. https://doi.org/10.1016/j.jaci.2022.04.012.

43. Schmitt, M., Schewe, M., Sacchetti, A., Feijtel, D., van de Geer, W.S., Teeuwssen, M., Sleddens, H.F., Joosten, R., van Royen, M.E., van de Werken, H.J.G., et al. (2018). Paneth Cells Respond to Inflammation and Contribute to Tissue Regeneration by Acquiring Stem-like Features through SCF/c-Kit Signaling. Cell Rep. *24*, 2312–2328.e7. https://doi.org/10.1016/j.celrep.2018.07.085.

44. Rothenberg, M.E., Nusse, Y., Kalisky, T., Lee, J.J., Dalerba, P., Scheeren, F., Lobo, N., Kulkarni, S., Sim, S., Qian, D., et al. (2012). Identification of a Ckit+ Colonic Crypt Base Secretory Cell that Supports Lgr5+ Stem Cells in Mice. Gastroenterology *142*, 1195–1205.e6. https://doi.org/10.1053/j.gastro.2012.02.006.

45. Haber, A.L., Biton, M., Rogel, N., Herbst, R.H., Shekhar, K., Smillie, C., Burgin, G., Delorey, T.M., Howitt, M.R., Katz, Y., et al. (2017). A single-cell survey of the small intestinal epithelium. Nature *551*, 333–339. https://doi.org/10.1038/nature24489.

46. Turtoi, E., Jeudy, J., Henry, S., Dadi, I., Valette, G., Enjalbal, C., and Turtoi, A. (2023). Analysis of polar primary metabolites in biological samples using targeted metabolomics and LC-MS. STAR Protoc. *4*, 102400. https://doi.org/10.1016/j.xpro.2023.102400.

47. Gerbe, F., van Es, J.H., Makrini, L., Brulin, B., Mellitzer, G., Robine, S., Romagnolo, B., Shroyer, N.F., Bourgaux, J.-F., Pignodel, C., et al. (2011). Distinct ATOH1 and Neurog3 requirements define tuft cells as a new secretory cell type in the intestinal epithelium. J. Cell Biol. *192*, 767–780. https://doi.org/10.1083/jcb.201010127.

## STAR★METHODS

### KEY RESOURCES TABLE

| REAGENT or RESOURCE | SOURCE | IDENTIFIER |
|---|---|---|
| Antibodies | | |
| Rabbit polyclonal anti Dclk1 | Abcam | Cat# Ab31704; RRID: AB_873537 |
| Sheep polyclonal to Dclk1 | R&D Systems | Cat# AF7138; RRID: AB_10973467 |
| Rabbit monoclonal [EPR16651] to Gata3 | Abcam | Cat# Ab199428; RRID: AB_2819013 |
| Rabbit polyclonal to Retnlb | Antibodies online | Cat# ABIN465494 |
| Mouse monoclonal to Insm1 | Santa Cruz | Cat# sc-271408; RRID: AB_10607955 |
| Rabbit polyclonal to Lysozyme | Dako | Cat# A0099; RRID: AB_2341230 |
| Rabbit polyclonal to Granzyme-B antibody | Abcam | Cat# Ab4059; RRID: AB_304251 |
| Rat monoclonal to Cd45 | Novus | Cat# NB100-77417; RRID: AB_1083776 |
| Rat monoclonal to MCPT-1 (mMCP-1) Antibody | eBioscience/ThermoFisher | Cat# 14-5503-82; RRID: AB_10854869 |
| Goat polyclonal to C-Kit antibody | R&D Systems | Cat# AF1356; RRID: AB_354750 |
| Rabbit polyclonal to Collagen V antibody | Gift From Patricia Simon Assmann | N/A |
| Rat anti mouse Siglec-F, PE-conjugated | BD pharmigen | Cat# 552126; RRID: AB_394341 |
| Rat anti mouse EpCam, APC-conjugated | eBioscience/ThermoFisher | Cat# 17-5791-82; RRID: AB_2716944 |
| Rabbit monoclonal to 5-Lipoxygenase Antibody (5Alox) | Novus | Cat# NBP3-161-50 |
| Goat polyclonal to FLAP (Alox5Ap) Antibody | Novus | Cat# NB300-891; RRID: AB_2227081 |
| Rabbit polyclonal to cyclooxygenase 2 antibody | Abcam | Cat# Ab225273 |
| Rabbit polyclonal to Prostaglandin D Synthase (hematopoietic-type) Antibody | Cayman chemical | Cat# 160013; RRID: AB_10080037 |
| Rabbit monoclonal to CD63 antibody | Abcam | Cat# Ab217345; RRID: AB_2754982 |
| Mouse monoclonal to Estrogen Receptor alpha antibody | Santa Cruz | Cat# Sc-8002; RRID: AB_627558 |
| Rabbit Monoclonal Anti-TBX21 (T-bet) | Clinisciences | Cat# AC-0240A |
| Rabbit Anti-Rorgt (clone EPR20006) | Abcam | Cat# ab207082; RRID: AB_2889310 |
| Rat Anti-mCD45-FITC (clone 30-F11) | BD Biosciences | Cat# 553080; RRID: AB_394610 |
| Rat Anti-mCD45-APC (clone 30-F11) | BD Biosciences | Cat# 559864; RRID: AB_398672 |
| Rat Anti-mCD45- APC-eF780 (clone 30-F11) | eBioscience/ThermoFisher | Cat#47-0451-80; RRID: AB_1548790 |
| Rat Anti-mCD19- BV650 (clone 1D3) | BD Biosciences | Cat# 563235; RRID: AB_273808 |
| Rat Anti-mCD19- V450 (clone 1D3) | BD Biosciences | Cat# 560375; RRID: AB_164526 |
| Hamster Anti-CD11c- PE (clone HL3) | BD Biosciences | Cat#557401; RRID: AB_396684 |
| Rat Anti-mCD11b-BV605 (clone M1/70) | BD Biosciences | Cat# 563015; RRID: AB_2737951 |
| Rat Anti-mCD4- BV711 (clone RM4-5) | BD Biosciences | Cat#563726; RRID: AB_2738389 |
| Rat Anti-mCD4- BV786 (clone RM4-5) | BD Biosciences | Cat# 563727; RRID: AB_2728707 |
| Hamster Anti-mTCRβ- FITC (clone H57-597) | BD Biosciences | Cat# 553170; RRID: AB_394682 |
| Hamster Anti-mTCRβ- AF594 (clone H57-597) | Biolegend | Cat# 109238; RRID: AB_2563324 |
| Hamster Anti-mTCRβ- V450 (clone H57-597) | BD Biosciences | Cat# 560706; RRID: AB_1727576 |

(Continued on next page)

*Continued*

| REAGENT or RESOURCE | SOURCE | IDENTIFIER |
|---|---|---|
| Rat Anti-mCD8α- APC-R700 (clone 53-6.7) | BD Biosciences | Cat#564983; RRID: AB_2739032 |
| Rat Anti-mCD8b- PE (clone H35–17.2) | BD Biosciences | Cat# 550798; RRID: AB_393887 |
| Rat Anti-mCD117- BV711 (clone2B8) | BD Biosciences | Cat# 563160; RRID: AB_272251 |
| Rat Anti-mCD117- PECF594 (clone2B8) | BD Biosciences | Cat# 562417; RRID: AB_11154233 |
| Rat Anti-mSca-1- FITC (clone D7) | BD Biosciences | Cat# 557405; RRID: AB_396688 |
| Rat Anti-mCD127- PE (clone A7R34) | eBioscience/ThermoFisher | Cat# 12-1271-82; RRID: AB_465844 |
| Hamster Anti-mKLRG1- PeCy7 (clone 2F1) | eBioscience/ThermoFisher | Cat# 25-5893-82; RRID: AB_1518768 |
| Anti-mGATA3- V450 (clone L50-823) | BD Biosciences | Cat# 563349; RRID: AB_2738152 |
| Mouse Anti-mRoRγt- BV650 (clone Q31378) | BD Biosciences | Cat# 564722; RRID: AB_2738915 |
| Mouse Anti-mTbet- PE (clone 4B10) | eBioscience/ThermoFisher | Cat#12-5825-82; RRID: AB_925761 |
| Rat Anti-mF4/80- PeCy7 (clone BM8) | eBioscience/ThermoFisher | Cat# 25-4801-82; RRID: AB_469653 |
| Rat Anti-mLy6C- V450 (clone HK1.4) | eBioscience/ThermoFisher | Cat# 48-5932-82; RRID: AB_10805519 |
| Rat Anti-mLy6G- PE-CF594 (clone 1A8) | BD Biosciences | Cat# 562700; RRID: AB_2737730 |
| Hamster Anti-mFceRIα - APC (clone MAR-1) | eBioscience/ThermoFisher | Cat# 17-5898-80; RRID: AB_10717073 |
| Live/Dead V506 | eBioscience/ThermoFisher | Cat#65-0866-14 |
| Donkey Anti-Rabbit IgG H&L (Alexa Fluor® 555) | Abcam | Cat# ab150074; RRID: AB_2715537 |
| Donkey Anti-Rabbit IgG H&L (Alexa Fluor® 647) | Abcam | Cat# ab150075; RRID: AB_2752244 |
| Donkey Anti-Rat IgG H&L (Alexa Fluor® 647) preadsorbed | Abcam | Cat# ab150155; RRID: AB_2813835 |
| Donkey Anti-Rat IgG H&L (Alexa Fluor® 488) preadsorbed | Abcam | Cat# ab150153; RRID: AB_2737355 |
| Donkey Anti-Mouse IgG H&L (Alexa Fluor® 555) | Abcam | Cat# ab150106; RRID: AB_2857373 |
| Donkey Anti-Mouse IgG H&L (Alexa Fluor® 488) | Abcam | Cat# ab150105; RRID: AB_2732856 |
| Donkey Anti-Sheep IgG H&L (Alexa Fluor® 488) | Abcam | Cat# ab150177; RRID: AB_2801320 |
| Donkey Anti-Goat IgG H&L (Alexa Fluor® 555) | Abcam | Cat# ab150130; RRID: AB_2715537 |
| **Chemicals, peptides, and recombinant proteins** | | |
| Neostigmine Bromide | Sigma-Aldrich | Cat# N2001 |
| Acetylcholine chloride | Sigma-Aldrich | Cat# A6625 |
| Atropine | Sigma-Aldrich | Cat# A0132 |
| Mecamylamine hydrochloride | Sigma-Aldrich | M9020 |
| RPMI culture medium | Life Technologies | Cat# 21875-034 |
| DMEM culture medium | Life Technologies | Cat# 21969-035 |
| FBS | Sigma-Aldrich | Cat# F7524 |
| Dispase | Corning | Cat# 354235 |
| DNAse 1 | Sigma-Aldrich | Cat# 11284932001 |
| 7-aminoactinomycin D | Life Technologies | Cat# A1310 |
| HBSS 10X w/o Ca2+/Mg2+ | GIBCO | Cat# 14185-045 |
| HBSS 1X avec Ca2+/Mg2+ | GIBCO | Cat# 14025-050 |

(*Continued on next page*)

*Continued*

| REAGENT or RESOURCE | SOURCE | IDENTIFIER |
|---|---|---|
| PBS 1X (Ca2+ et Mg2+ free) | Eurobio | Cat# CS1PB01-01 |
| LiberaseTM | Roche | Cat# 5401119001 |
| EDTA | Sigma-Aldrich | Cat# 03690-100 |
| DTT | Sigma-Aldrich | Cat# 43816-50 |
| Vetflurane 1000 mg/g | Virbac | GTIN 03597132002653 |
| Buprecare 0.3 mg/ml | Axience | GTIN 03760087151244 |
| Tamoxifene | Sigma-Aldrich | Cat# T5648 |
| Laocaine 16mg | MSD | GTIN: 05017363520132 |
| Trizol reagent | Thermo Fisher | Cat# 15596026 |
| Buprenorphine | Sigma-Aldrich | Cat#B9275 |
| Hematoxylin Solution, Gill No. 3 | Sigma-Aldrich | Cat#GHS316 |
| SIGMAFAST™ 3,3′-Diaminobenzidine tablets | Sigma-Aldrich | Cat# D4293 |
| N-Histofine | Nichirei Biosciences | 414151F |
| **Critical commercial assays** | | |
| ATPLite kit | Perkin Elmer | Cat# 6016943 |
| RNeasy Micro Kit | Qiagen | ID: 74004 |
| RNeasy Mini Kit | Qiagen | ID: 74104 |
| Transcriptor First Strand cDNA Synthesis Kit | Roche lifescience | Cat# 04896866001 |
| LightCycler® 480 SYBR Green I Master | Roche lifescience | Cat# 04887352001 |
| RNAscope™ Multiplex Fluorescent V2 Assay | ACD a biotechne brand | Cat# 323270 |
| Fix/perm kit FOXP3/transcription factor staining buffer set | eBioscience/ThermoFisher | Cat# 00-5523-00 |
| Arcturus RiboAmp Plus kit | Thermo Fisher | Cat# KIT0501 |
| **Experimental models: Organisms/strains** | | |
| Mouse strain $Chat^{tm1Mlt}$ | Lecomte et al.[22] | Provided by S. Berrard |
| Mouse strain Tg(Vil1-cre/ERT2)23Syr | El Marjour et al.[23] | Provided by S. Robine |
| Mouse strain $Chat^{LoxP/LoxP}$; $VillinCre^{ERT2/+}$ | This study | N/A |
| *Heligmosomoides polygyrus* | Provided by R. Maizels | Provided by R. Maizels |
| **Oligonucleotides** | | |
| *Chat* | Forward TTTGCAGCCAGCCTCATCTC<br>Reverse TATGGCCAGGAAGCCGGTAT | Eurofins |
| *VAChT* | Forward GGCCTCGCTACCCCACAGAA<br>Reverse CCCAGGCCAATAAGCAGCGG | Eurofins |
| *Pou2f3* | Forward GAGGGAATGATGAGCCCACT<br>Reverse GTGAAGCCTAGCTTAATGCGTC | Eurofins |
| *Alox5* | Forward TCTTCCTGGCACGACTTTGCTG<br>Reverse GCAGCCATTCAGGAACTGGTAG | Eurofins |
| *Alox5ap* | Forward GTTCTTTGCCCACAAGGTGGAG<br>Reverse TGCAGTCCAGAGTACCACAAGG | Eurofins |
| *Ptgs1* | Forward GAATGCCACCTTCATCCGAGAAG<br>Reverse GCTCACATTGGAGAAGGACTCC | Eurofins |
| *Ptgs2* | Forward TCAATGAGTACCGCAAACGC<br>Reverse AGGGTACAGTTCCATGACATCG | Eurofins |
| *Hpgds* | Forward GAATAGAACAAGCTGACTGGC<br>Reverse AGCCAAATCTGTGTTTTTGG | Eurofins |
| *Ltc4s* | Forward CCTACAGGTGATCTCTGCACGA<br>Reverse TGGCGAGGAACAGCGGAAAGTA | Eurofins |
| *Sucnr1* | Forward CCATCTCTGACTTTGCTTTCCTG<br>Reverse GTGTAGAGGTTGGTGTGAAGCAC | Eurofins |
| *Dclk1* | Forward CAGCCTGGACGAGCTGGTGG<br>Reverse TGACCAGTTGGGGTTCACAT | Eurofins |

(*Continued on next page*)

| *Continued* | | |
|---|---|---|
| REAGENT or RESOURCE | SOURCE | IDENTIFIER |
| *Il25* | Forward CCTGTCAGGCAGGGGTAGTA<br>Reverse CCAAGAATGCAACAGCCTG | Eurofins |
| *Villin* | Forward: CAAGCCTGGCTCGACGGCC<br>Reverse: CGCGAACATCTTCAGGTTCT | Eurofins |
| *Chat* (exon8) | Forward: CCCGTTTTCCTTCCAGCCAA<br>Reverse: CGCGAACATCTTCAGGTTCT | Eurofins |
| *Gapdh* | Forward GGAGCGAGACCCCACTAACA<br>Reverse ACATACTCAGCACCGGCCTC | Eurofins |
| *Hprt* | Forward GCAGTACAGCCCCAAAATGG<br>Reverse GGTCCTTTTCACCAGCAAGCT | Eurofins |
| Software and algorithms | | |
| NdpView.V2 | Hamamatsu Photonics | https://www.hamamatsu.com |
| Zen blue edition. V3.2 | Zeiss | https://www.zeiss.com/microscopy/fr/produits/logiciel/zeiss-zen.html |
| Photoshop CS6. V13.0 | Adobe | https://www.adobe.com/fr/products/photoshop.html |
| Prism10.1 | GraphPad | https://www.graphpad.com |
| LightCycler 480 V1.5 | Roche | https://lightcycler-software.software.informer.com |
| ImageJ. V1.8 | Wayne Rasband (NIH) | https://imagej.net/ij/download.html |
| FlowJo, LLC V9 | Tree Star; Ashland, OR | https://www.flowjo.com/ |
| Biorender | BioRENDER | https://www.biorender.com/ |
| Other | | |
| RNAscope™ Probe- Mm-Chat-C2 | Advanced Cell Diagnostics | Cat# 408731-C2 |
| RNAscope™ Probe- Mm-Sucnr1-C3 | Advanced Cell Diagnostics | Cat# 437721-C3 |
| RNAscope™ Probe- Mm-Ltc4s-C3 | Advanced Cell Diagnostics | Cat# 1046751-C3 |

## RESOURCE AVAILABILITY

### Lead contact

Further information and requests for resources and reagents should be directed to and will be fulfilled by the lead contact, Philippe Jay (philippe.jay@igf.cnrs.fr).

### Materials availability

All stable reagents generated in this study are available from the lead contact with a completed materials transfer agreement.

### Data and code availability

- All data reported in this paper will be shared by the lead contact upon request.
- This paper does not report original code.
- Any additional information required to reanalyze the data reported in this paper is available from the lead contact upon request.

## EXPERIMENTAL MODEL AND STUDY PARTICIPANT DETAILS

### Mouse models

The conditional *Chat*$^{LoxP/LoxP}$ allele, (Chat$^{tm1Mlt}$,[22]) was kindly provided by Sylvie Berrard and crossed with the intestinal epithelium-specific, tamoxifen-inducible, *Villin-Cre*$^{ERT2}$ mouse strain (Tg(Vil1-cre/ERT2)23Syr.[23] All the mice were bred and maintained in an SOPF animal facility. All animal experiments were conducted in accordance with the French Ministry for Education and Research, regarding the care and use of animals for experimental procedures, under the 2022020111554986, and 2019031411197134 Apafis references, according the ARRIVE Guidelines. Male and female mice were analyzed between 8 and 12 weeks of age. Cohorts of controls and deficient mice were obtained from littermates. No statistical method was used to predetermine sample size and the experiments were not randomized. Unless otherwise stated, the investigators were not blinded to allocation during experiments and outcome assessment.

## METHOD DETAILS

### Animal procedures

Cre-mediated recombination was achieved with a daily intraperitoneal injection of 1 mg of tamoxifen (Sigma), for 5 consecutive days. For *H. polygyrus* infection experiments, mice were inoculated orally with 200 infective L3. Infection parameters were monitored according to the numbers of fecal eggs, or numbers of intestinal adult worms from day 10 to day 40-post infection. Samples of luminal intestinal contents were obtained from naive or infected mice, according to the following surgery procedure. After 4 h of fasting, mice received subcutaneously a single dose of buprenorphine (0.1 mg/kg body weight), and were anesthetized 30 min later with 3% isoflurane. After local lidocaine treatment, the abdominal cavity was opened, and an intestinal loop was ligatured. This intestinal loop was filled with up to 400 μL of a PBS wash solution containing 5 μM of Neostigmine Bromide (Sigma). The loop content was recovered 30 min later, after which mice were euthanized.

### Collection of adults *H. polygyrus* and *in vitro* culture for treatment with cholinergic drugs

Adult *H. polygyrus* worms were collected from the intestinal lumen of infected mice at 14 dpi to evaluate *ex vivo* the effects of cholinergic drugs on egg release. Worms were washed with PBS containing penicillin (5U/ml)/streptomycin (5 μg/ml)/gentamicin (1%), counted, and 20 to 25 female worms per well were incubated at 37°C, 5% CO2, in 200μL RPMI containing antibiotics. Adult *H. polygyrus* worms were then treated with the following drugs: acetylcholine chloride (Sigma, A6625) at 10μM, atropine (Sigma, A0132), and mecamylamine hydrochloride (Sigma, M9020) at 5μM. After 24h, culture media were mixed with a saturated NaCl solution (in a 50/50 volume ratio) to count the eggs. For global worm fitness evaluation, worms were collected from mice at 20 and 40 dpi, washed in PBS, and directly used as substrate for ATP quantification, using the ATPLite kit (6016943; PerkinElmer), according to the manufacturer's instructions.

### Cell sorting experiments

Single intestinal epithelial cells were obtained from small intestines after incubation in ice-cold 30mM EDTA (Sigma) in HBSS pH 7.4 (Life Technologies) for 20 min. Tissues were then vigorously shaken in DMEM (Life Technologies) supplemented with 10% FBS (Sigma), with 100μL of Dispase (Corning), and 100μL of DNase I at 2,000 Kunitz (Sigma). After filtration on a 40 μm mesh, single cell suspensions were incubated with phycoerythrin rat anti-mouse Siglec-F antibody (BD Pharmigen, 552126), and FITC rat anti mouse EpCam antibody (17-5791-82, ebiosciences) for 30 min at 4°C, and washed with HBSS and resuspended in appropriate volume of HBSS pH 7.4 supplemented with 5% FBS before staining with 7-aminoactinomycin D (Life Technologies) to exclude dead cells. Siglec-F+ live cells were sorted using a FACSAria (Becton Dickinson), directly in RLT lysis buffer (Qiagen) for subsequent RNA extraction, or methanol for ACh quantification assays. After removing epithelial cell fractions as described above, the remaining tissues were proceeded for *lamina propria* cell preparation. Tissues were washed twice with RPMI 1640 (Life technologies) supplemented with 5% FCS, and minced into small pieces and digested with a solution composed with RPMI 1640, 100μg/mL of Liberase TM (Roche) and 50μg/mL of DNase I (Sigma-Aldrich) for 30 min at 37°C under gentle shaking. Supernatants were filtered onto a 40μm mesh. After a 5 min centrifugation at 2000RPM, 4°C, cell pellets were resuspended in 500μL PBS-5% FCS-2mM EDTA for further stainings.

### Immunophenotyping and flow cytometric analysis

Cells isolated from peripheral lymph nodes, mesenteric lymph nodes, spleen and intestinal tissue (*lamina propria* and intra-epithelial lymphocytes) were stained with Live/dead fixable viability dye (Ebioscience/Thermofisher) together with the appropriate conjugated anti-TCRβ, CD45, CD19, CD4, CD8, CD11b, Ly6G, Ly6C, FcεRIa, F4/80, CD117 (eBioscience/Thermofisher or Becton Dickinson) at a 1:200 dilution (mAb), in a total volume of 50–100 μls as previously described. Cells were incubated in the dark for 20 min in PBS containing 2% FBS at 4°C and then washed once in the same medium at 300 g for 5 min prior to be evaluated by flow cytometry. For ILC staining, cells were stained with a lineage cocktail and lineage-negative $CD45^+$ cells were assessed for expression of CD127, and intracellular expression of Gata-3 (clone L50-823), RoRγt and Tbx21 was performed following fixation/permeabilization (eBioscience/Thermofisher). Stained cells were assessed by flow cytometry (LSR Fortessa, Becton Dickinson, San Jose, CA) and a minimum of 10,000 events were recorded for each staining. Data analyses were performed using FlowJo software (Tree Star, Ashland, OR).

### ACh quantification

Known numbers of sorted tuft cells (recovered in pure methanol) and intestinal lavage samples (0.2 mL, immediately mixed with 0.8 mL pure methanol), were kept at −80C° for 48h to allow protein precipitation. Next, 2-morpholinoethansulfonic acid (Cat. # 341–01622; Dojindo, Tokyo, Japan) was added as quality control (QC) standard at 0.5 μM final concentration (FC) and the samples were then supplemented with formic acid to 1% FC. The samples were vortexed vigorously for 1 min and then centrifuged for 10 min at 20.000xg to precipitate proteins. A volume of 0.9 mL supernatant was then loaded on the Captiva EMR plate (Agilent, Santa Clara, USA, Cat.# 5190-1001) assembled on the Vacuum Manifold (Agilent, Cat.# A796) together with the Deep Well collection plate (Agilent, Cat.# A696001000). The flow-through fractions were first dried using a Speedvac concentrator (Thermo Scientific, Cat.#

SPD2030) and then resuspended in 200 μL of water (Biosolve BV, Cat. # 0023214102BS). Calibration curve was established by diluting defined amounts of ACh in the matrix (generated by intestinal lavage of KO mice). Protein quantities from each sample were measured using the BCA kit (Thermo Scientific) according to manufacturer's recommendations. Briefly, protein pellets were solubilized in 0.3 mL of 2% sodium dodecyl sulfate solution (Sigma Aldrich, Cat. # 05030) in water. Two μL of sample was then used to estimate the protein content of each sample.

One microliter of resuspended Captiva flow-through fraction or 1 μL of standard were injected on LC-MS systems consisting of UHPLC (Agilent, 1290 Infinity II Biocompatible) coupled to triple quadrupole MS (Agilent, 6495C). The samples were analyzed using in MRMs acquisition mode. Following transitions were used to quantify ACh: 147.1 → 43.0, 147.1 → 88.1, 147.1 → 87.1 (positive mode). The transitions for the 2-morpholinoethansulfonic acid were: 196.2 → 100.0 (positive) and 194 → 80.15 (negative). The retention times were set as follows: 2.04 min and 8 min, for 2-morpholinoethansulfonic acid and ACh respectively. The analytical column was UPLC Discovery column HS F5-3 (Cat. # 567503-U; Sigma Aldrich). Mobile phases were composed as follows: A: 99.9% water (Biosolve BV, Cat. # 0023214102BS), 0.1% formic acid (Sigma Aldrich, Cat. # 33015); B: 99.9% acetonitrile (Biosolve BV, Cat.# 001204102BS), 0.1% formic acid. The gradient was: 0 min (100% A), 2 min (100% A), 5 min (75% A), 11 min (65% A), 15 min (5% A), 25 min (5% A), 25.10 min (100% A), 35 min (100% A). Flow rate was 0.25 mL/min and the temperature column was set to 40°C. The following source parameters were used: Gas Temperature: 150°C; Gas Flow: 11 L/min; Nebulizer: 40 psi; Sheath Gas Temperature: 400°C; Sheath Gas Flow: 12 L/min; Capillary Voltage (neg. mode): 4000 V; Capillary Voltage (pos. mode): 4000 V; Nozzle Voltage: 500 V; IFunnel High Pressure RF Pos: 100 V Neg: 50 V; IFunnel Low Pressure RF Pos: 100 V Neg: 50 V.

Following the analysis, peak integration was conducted using Agilent Masshunter Quantitative Analysis software (version 10.1.733.0). The absolute quantification of ACh was calculated as previously demonstrated, by applying the peak area from each sample to the ACh calibration curves.[46]

### RNA extraction and PCR

Total RNAs from intestinal tissues were isolated using TRIzol (Life Technologies). RNeasy Micro and Mini Kit columns (Qiagen) were also used for RNA purification from cell-sorted experiments. Sorted-cell RNA were further amplified using the Arcturus RiboAmp Plus kit (ThermoFischer Scientific, KIT0501) according to the manufacturer's instructions. Reverse transcription was performed with 1 μg of purified RNA using Transcriptor First Strand cDNA synthesis KIT (Roche) according to the manufacturer's instructions. Real-time quantification was performed in triplicate with a LightCycler480 (Roche) using LightCycler 480 SYBR Green I Master (Roche) on 5ng of RT product using the average Ct of *Gapdh* and *Hprt* as internal loading controls, and the ΔΔCt method was used for calculating relative expression.

### Fluorescent immunohistochemistry or *in situ* hybridization on paraffin-embedded tissue

Tissue dissection, fixation, and immunohistochemistry on thin sections of paraffin-embedded tissue were performed essentially as described previously.[47] Epitope retrieval was achieved by boiling in 10 mM in sodium citrate (pH 6.4) during 20 min. Primary antibodies used in this study were incubated ON at 4°C, and are listed in the key resource table. Slides were then washed twice with 0.1% PBS-Tween (Sigma-Aldrich) before incubation with fluorescent dyes-conjugated secondary antibodies (Jackson ImmunoResearch Laboratories, Inc.) and DAPI at 2 μg/mL (Sigma-Aldrich) in TBS–Triton X-100 0.1% (Sigma-Aldrich), or HRP-conjugated secondary antibodies, revealed with DAB (Sigma). Slides were mounted in FluoroMount (Sigma) or Pertex (Histolab), for fluorescent or visible imaging, respectively. For mRNA *in situ* hybridisation, tissues were hybridised with a probe targeting the *Chat* mRNA (Cat No. 408731, ACDBio), *SucnR1* (Cat No 437721), and *Ltc4s* (Cat No 1046751). Slides were processed according to the manufacturer's instructions until probe revelation, after which immunofluorescence detection of Dclk1 was performed following the methodology described above.

### Microscopy and imaging

Fluorescent pictures were acquired at room temperature on an AxioImager Z1 microscope (Carl Zeiss, Inc.) equipped with a camera (AxioCam MRm; Carl Zeiss, Inc.), EC Plan Neofluar (5X NA 0.16; 10X NA 0.3; 20X 0.5 NA; 100X NA 1.3) and Plan Apochromat (40X NA 0.95; 63X NA 1.4) lenses, apotome Slider system equipped with an H1 transmission grid (Carl Zeiss, Inc.), and Zen software (Carl Zeiss, Inc.). Post-treatment of pictures (level correction), annotations, and panel composition were performed using the Photoshop (Adobe) or Zen (Carl Zeiss) softwares. Bright-field immunohistochemistry pictures were taken at room temperature on an Eclipse 80i microscope (Nikon) with Plan Fluor (10X NA 0.3; 20X NA 0.5; 40X NA 0.75; and 60X NA 0.5–1.25) lenses (Nikon) and a digital camera (Q-Imaging Retiga 2000R with a Q-Imaging RGB Slider), with Q-Capture Pro software (Nikon). Stained slides were also imaged with a Nanozoomer device (Hamamatsu), visualised and annotated with the NdpView Software (Hamamatsu).

## QUANTIFICATION AND STATISTICAL ANALYSES

The Prism software was used for descriptive statistical analyses. After a normality distribution test, datasets were analyzed with either a two-tailed Student T test (normal distribution, pairwise comparisons) or with the non-parametric two-tailed Mann–Whitney U-test (nonnormal distribution, pairwise comparisons). For multiple comparisons, datasets were proceeded with one-way ANOVA analyses

(normal distribution, with Benjamini-Yekutieli post-hoc correction) or with Kruskal-Wallis analyses (nonnormal distribution, with Benjamini-Yekutieli post-hoc correction). For some analyses, multiple Mann-Whitney analyses were performed. Depending on the normality distribution, datasets are represented in each graph as individual values and bars corresponding to mean ± SD (normal distribution) or either as individual values and bars corresponding to median ± interquartile (nonnormal distribution). Details for statistical analyses are mentioned in each figure legend.

**Immunity, Volume *57***

## Supplemental information

# Tuft cell acetylcholine is released into the gut lumen to promote anti-helminth immunity

**Marième Ndjim, Imène Gasmi, Fabien Herbert, Charlène Joséphine, Julie Bas, Ali Lamrani, Nathalie Coutry, Sylvain Henry, Valérie S. Zimmermann, Valérie Dardalhon, Marta Campillo Poveda, Evgenia Turtoi, Steeve Thirard, Luc Forichon, Alicia Giordano, Claire Ciancia, Zeinab Homayed, Julie Pannequin, Collette Britton, Eileen Devaney, Tom N. McNeilly, Sylvie Berrard, Andrei Turtoi, Rick M. Maizels, François Gerbe, and Philippe Jay**

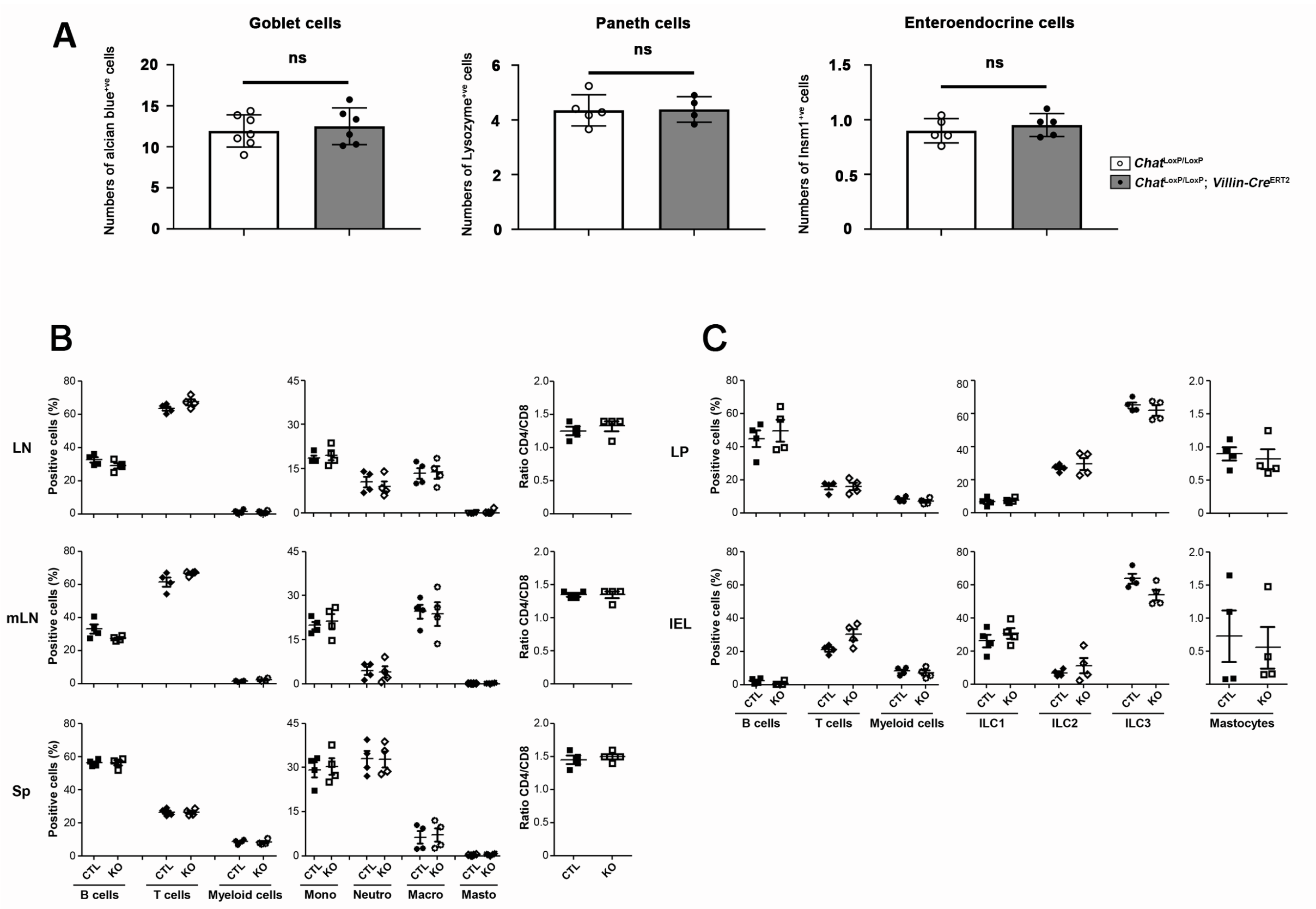


**Figure S1: Intestinal epithelial ChAT deficiency does not cause drastic changes in intestinal epithelium cellular composition and does not impact the immune landscape in the intestine and in distant lymphoid organs, related to Figure 2. (A)** Goblet, Paneth and enteroendocrine cells were counted in naive mice, 10 days after recombination of the *chat* locus. Alcian blue staining was used to highlight goblet cells and IHC against lysozyme and Insm1, to reveal Paneth and enteroendocrine cells, respectively. Data represent means ± SD of the biological replicates (n ranging from 4 to 7, ns = p > 0.05 after a Student T test). **(B)** The repartition of immune cells in control (*Chat*$^{LoxP/LoxP}$) and *Chat*-deficient (*Chat*$^{LoxP/LoxP}$;*Villin-Cre*$^{ERT2}$) mice was monitored by flow cytometry in lymph nodes (LN), mesenteric lymph nodes (mLN) and spleens (Sp). The presence of T (TCRβ+) including CD4/CD8 ratio, B (CD19+), total myeloid (CD11b+) cells as well monocytes (mono, CD11b+Ly6C+Ly6G-), neutrophils (neutro, CD11b+Ly6C+Ly6G+), macrophages (macro, CD11b+F4/80+), mastocytes (CD11b+CD117+FcεRIa+) was assessed by staining with fluorochrome-tagged antibodies. Each point represents one mouse and means ± SD are shown. (n = 4 mice per genotype). **(C)** Immune lineage cells in the *lamina propria* (LP) and intra-epithelial lymphocyte (IEL) of control *(Chat*$^{LoxP/LoxP}$) and *Chat*-deficient (*Chat*$^{LoxP/LoxP}$;*Villin-Cre*$^{ERT2}$) mice were monitored by flow cytometry after tissue dissociation. The percentage of T, B and myeloid cells were assessed within the CD45+ haematopoietic gate, and ILC subsets within the CD45+CD19-CD3-CD11b-CD127+ population. Representative dot plots are presented. Quantification of immune cells are shown; each point represents a mouse as means ± SD (n = 4 mice per genotype). Statistical analyses were performed using a Mann Whitney test and no significant differences were detected for all cell types between *Chat*$^{LoxP/LoxP}$ and *Chat*$^{LoxP/LoxP}$*;Villin-Cre*$^{ERT2}$ mice.

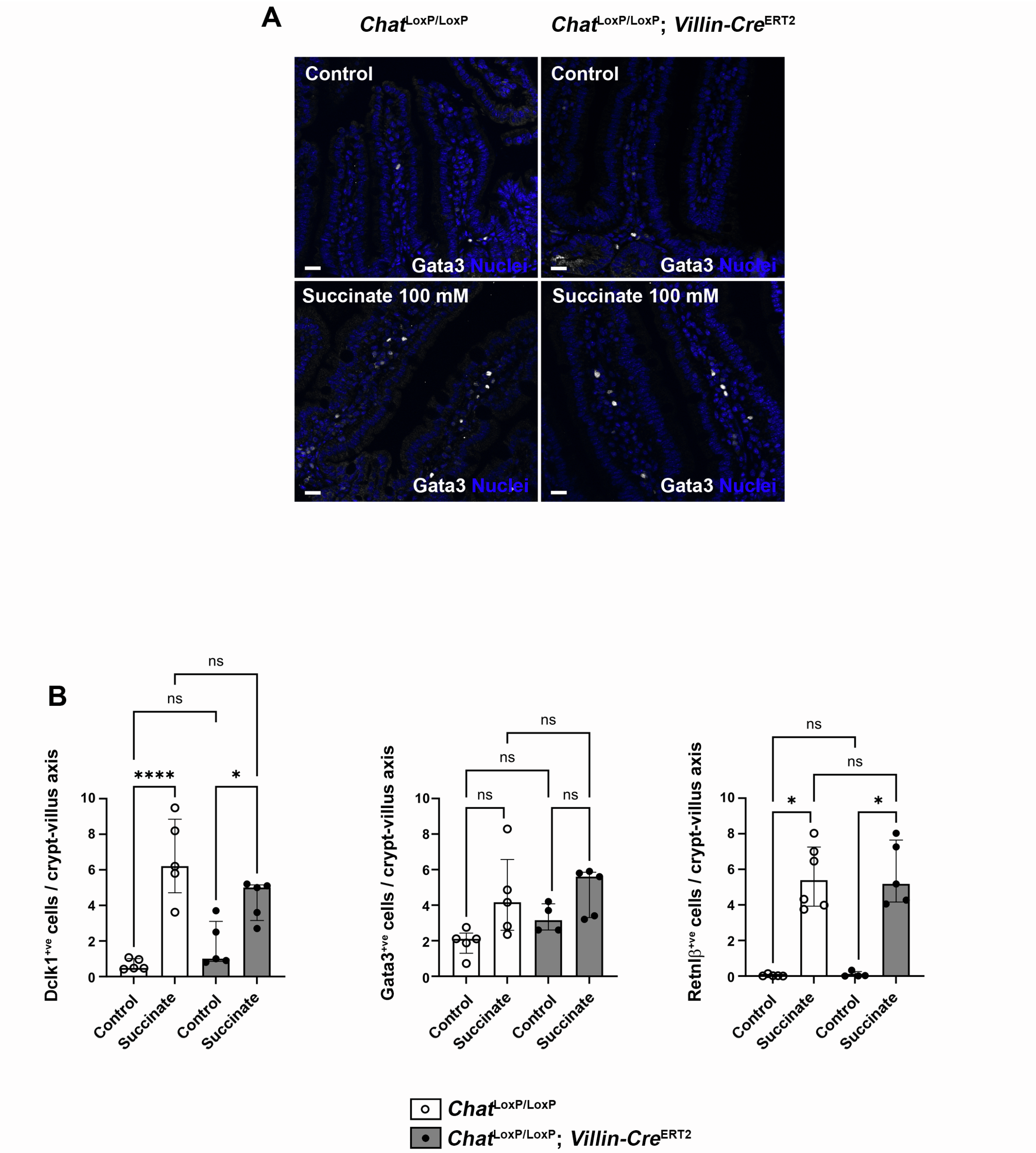


**Figure S2: ChAT-deficient mice are able to mount a type-2 immune response after a succinate-mediated tuft cell activation, related to Figure 4. (A)** Representative Gata3 staining (grey) by IHC for both *Chat*$^{LoxP/LoxP}$ and *Chat*$^{LoxP/LoxP}$;*Villin-Cre*$^{ERT2}$ mice, treated or not with 100 mM succinate for one week (scales: 10µm) **(B).** Quantification of type-2 immune response parameters in *Chat*$^{LoxP/LoxP}$ and *Chat*$^{LoxP/LoxP}$;*Villin-Cre*$^{ERT2}$ mice, treated or not with 100 mM succinate for one week, as assessed by counting Dclk1-expressing tuft cells (n=5 for each group), Gata3-expressing ILC2/Th2 cells (n=4 and 5), and Resistin-like beta-expressing goblet cells (n=4 and 6). Data represent median ± interquartile of the biological replicates (n= 4 to 6 for each condition, ns = $p > 0.05$, * = $p < 0.05$, **** = $p < 0.0001$ after Kruskal-Wallis analyse).

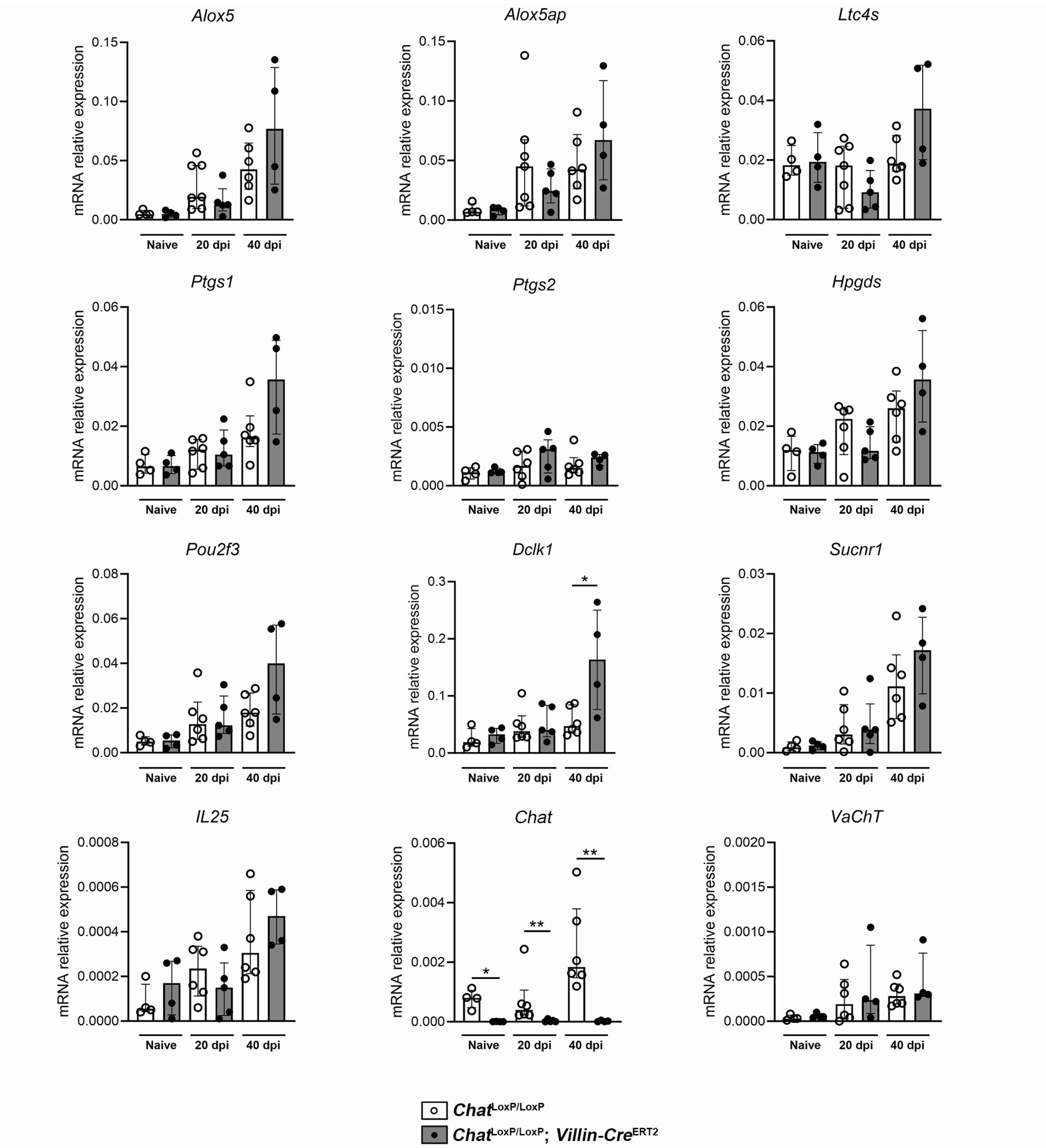


**Figure S3: Expression levels of transcripts related to leukotrienes and prostanoids biosynthesis pathways, tuft cells markers and alarmin function, and ACh synthesis and vesicular transport, related to Figure 4.** PCR was performed on mRNA extracted from enriched epithelial cell suspensions recovered from 4 to 7 tamoxifen-treated naive and *H. polygyrus*-infected *Chat*$^{LoxP/LoxP}$ control (white bar and circles) or *Chat*$^{LoxP/LoxP}$;*Villin-Cre*$^{ERT2}$ mice (grey bar and circles), 20 and 40 days post-infection. Data represent median ± interquartile of the biological replicates, * = $p < 0.05$, ** = $p < 0.01$, after a Mann Whitney test.

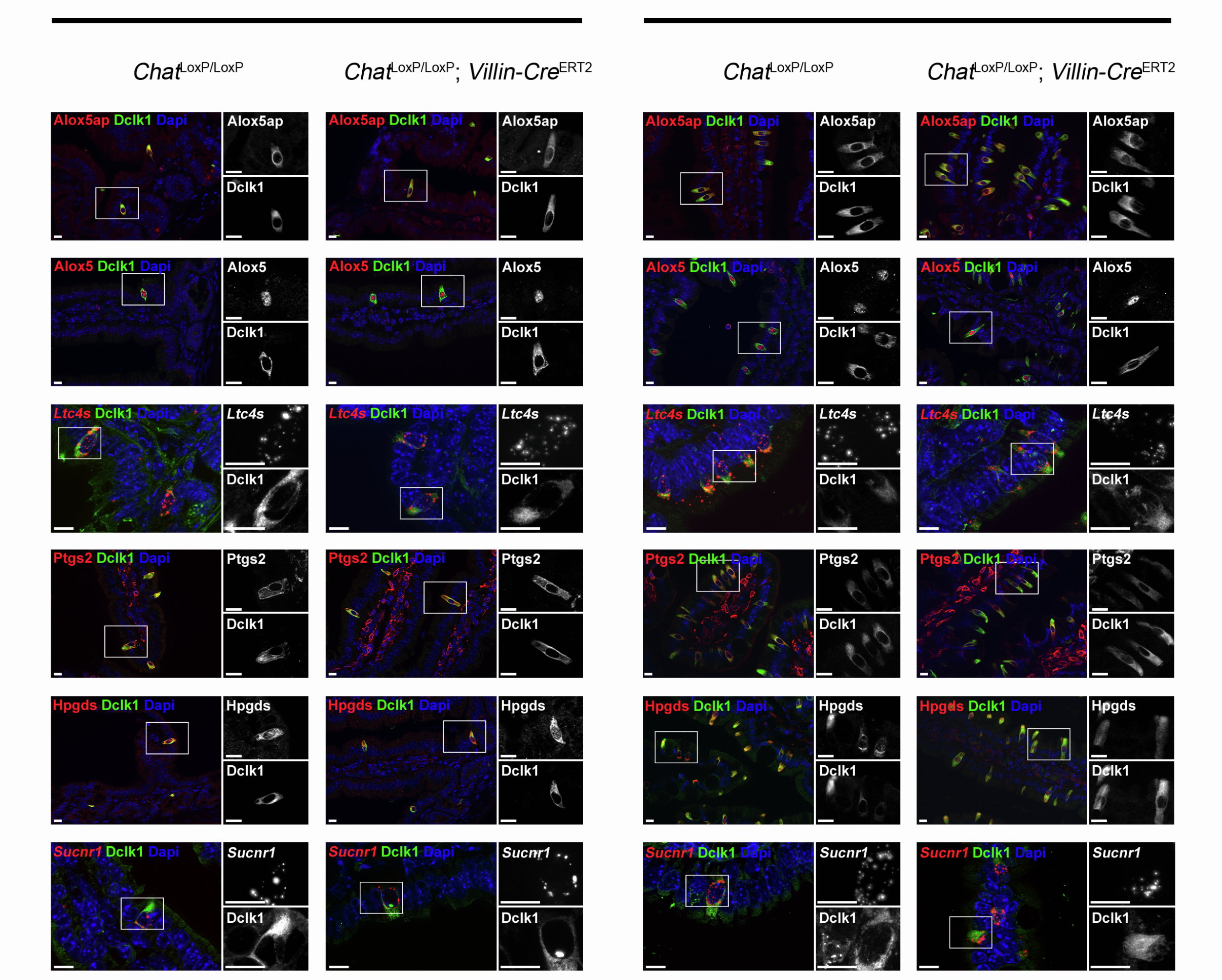


**Figure S4: ChAT deficiency does not impair expression of tuft cell specific, type-2 immune response-related proteins, related to Figure 4.** Representative double IHC, or RNAscope combined with IHC, for the tuft cell marker Dclk1 (green on overlays), leukotriene C4 biosynthesis (Alox, Alox5ap and *Ltc4s*), $PGD_2$ biosynthesis (Ptgs2, Hpgds), and succinate receptor (*Sucnr1*), in tamoxifen-treated naive and *H. polygyrus*-infected (20 days post-infection) $Chat^{LoxP/LoxP}$ and $Chat^{LoxP/LoxP}$;$Villin\text{-}Cre^{ERT2}$ mice (n=3 for each condition). Scales: 10µm.

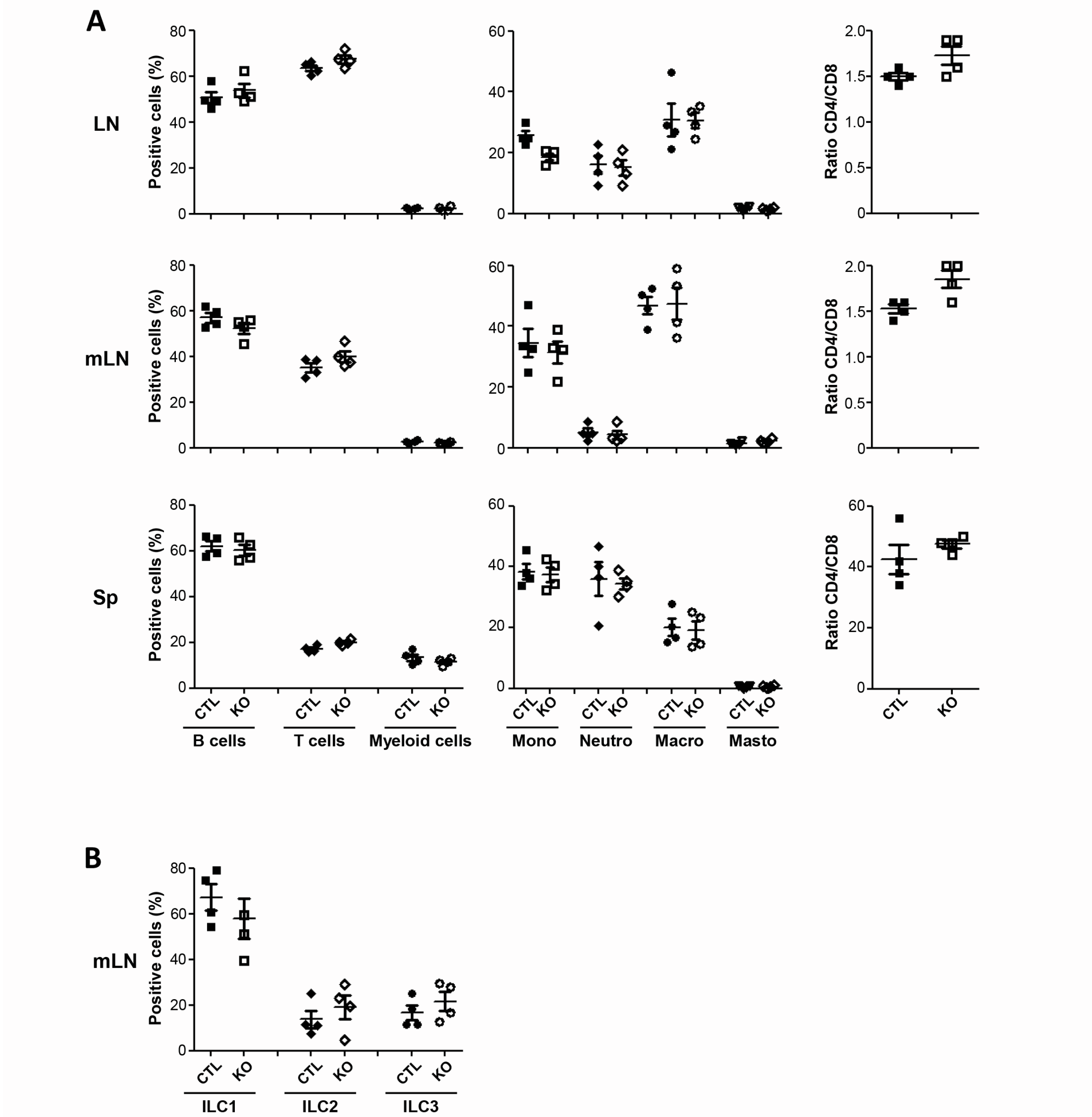


**Figure S5: Epithelial ChAT deficiency does not impact the immune landscape within the intestine and in distant lymphoid organs during infections, related to Figure 4. (A)** The repartition of immune cells in wild-type (*Chat*$^{LoxP/LoxP}$) and ChAT-deficient (*Chat*$^{LoxP/LoxP}$;*Villin-Cre*$^{ERT2}$) mice was monitored by flow cytometry in lymph nodes (LN), mesenteric lymph nodes (mLN) and spleens (Sp) following *H. polygyrus* infections. The presence of T (TCRβ+) including CD4/CD8 ratio, B (CD19+), total myeloid (CD11b+) cells as well as monocytes (mono, CD11b+Ly6C+Ly6G-), neutrophils (neutro, CD11b+Ly6C+Ly6G+), macrophages (macro, CD11b+F4/80+), mastocytes (CD11b+CD117+FcεRIa+) was assessed by staining with fluorochrome-tagged antibodies. Each point represents one mouse and means ± SD are shown. (n = 4 mice per genotype). **(B)** Immune lineage cells in the mesenteric lymph nodes (mLN) of *H. polygyrus*-infected control *(Chat*$^{LoxP/LoxP}$) and ChAT-deficient (*Chat*$^{LoxP/LoxP}$;*Villin-Cre*$^{ERT2}$) mice were monitored by flow cytometry after tissue dissociation. The percentage of ILC subsets were assessed within the CD45+CD19-CD3-CD11b-CD127+ population. Representative dot plots are presented. Quantification of immune cells are shown; each point represents a mouse as means ± SD (n = 4 mice per genotype). Statistical analyses were performed using a Mann Whitney -test and no significant differences were detected for all cell types between *Chat*$^{LoxP/LoxP}$ and *Chat*$^{LoxP/LoxP}$;*Villin-Cre*$^{ERT2}$ mice.

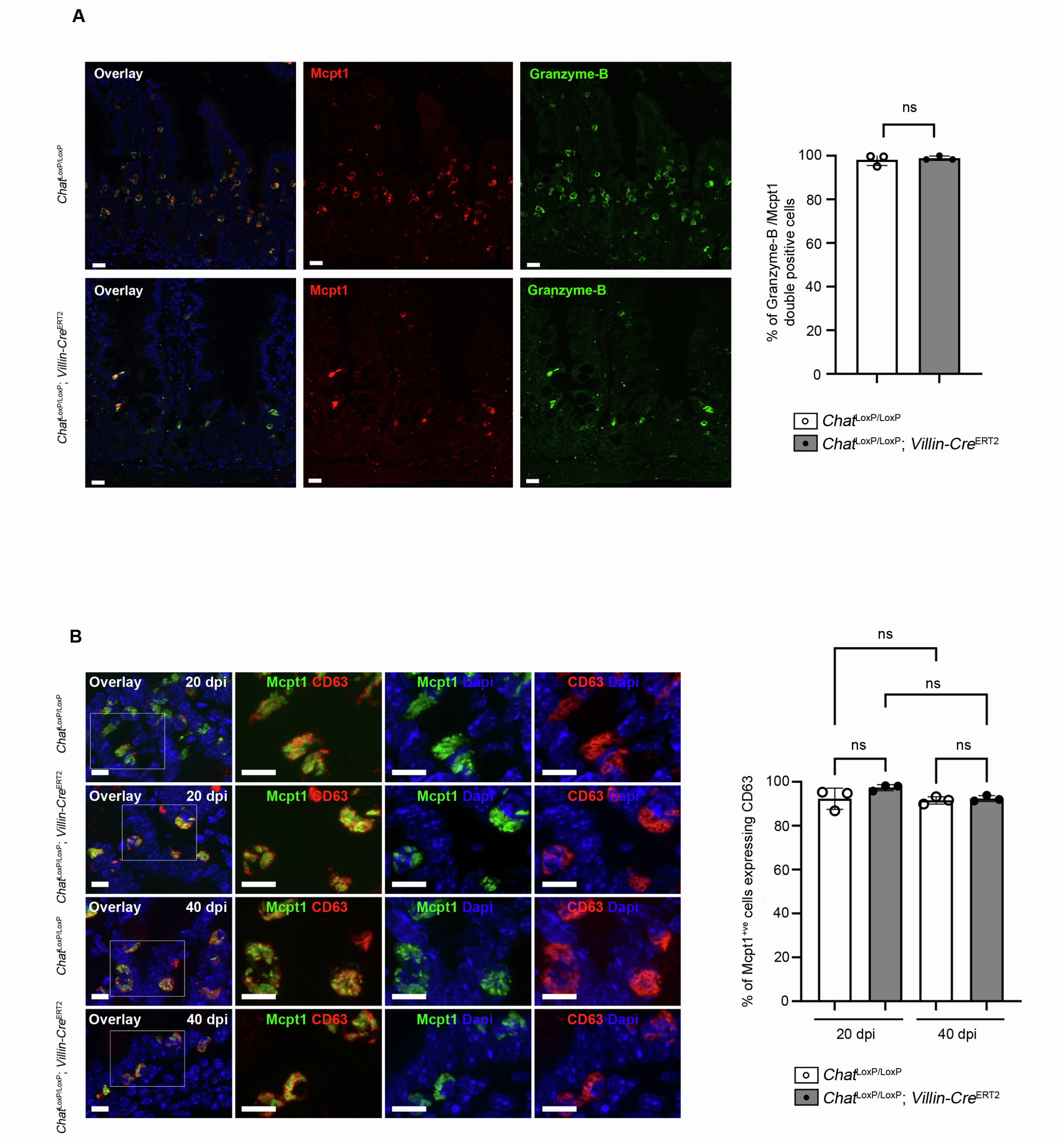


**Figure S6: ChAT deficiency does not impair mast cell marker expression and activation, related to Figure 5. (A)** Representative double IHC for Mcpt1 (red) and Granzyme-B (green), from 20 dpi mice. The graph represents the percentage of co-expression between these two markers. Data represent median ± interquartile of the biological replicates (for a total 318 and 783 counted mast cells in n=3 mice from both *Chat*$^{LoxP/LoxP}$ and *Chat*$^{LoxP/LoxP}$;*Villin-Cre*$^{ERT2}$ genotypes, ns = p > 0.05 after a Mann-Whitney test). **(B)** Representative immunostaining for Mcpt1 (green) and CD63 (red) in *Chat*$^{LoxP/LoxP}$ and *Chat*$^{LoxP/LoxP}$;*Villin-Cre*$^{ERT2}$ mice, at 20 and 40 dpi with *H. polygyrus*. (Scales: 10µm). Graph showing the percentage of co-expression between these two markers. Data represent means ± SD of the biological replicates, for a total of 469 and 324 counted mast (20 dpi), and 239 and 305 counted mast cells (40 dpi), in 3 mice of both *Chat*$^{LoxP/LoxP}$ and *Chat*$^{LoxP/LoxP}$;*Villin-Cre*$^{ERT2}$ genotypes, ns = p > 0.05 after ANOVA analyse.

| Mouse ID | Recovered volume of intestinal content (µl) |
|---|---|
| 1 | 20 |
| 2 | 18 |
| 3 | 8 |
| 4 | 9 |
| 5 | 13 |
| 6 | 18 |
| **Mean** | **14.33** |
| **SD** | **4.64** |

**Figure S7: Estimation of small intestinal luminal content volumes prior to surgeries, related to Figure 6.** The table shows recovered volumes of small intestinal contents prior to surgeries. Data were acquired from 6 mice, means and SD are shown.
