# Supplementary material for: Tuft cell acetylcholine is released into the gut lumen as an effector and regulator of type 2 immunity, and directly targets helminth parasites
Source: Immunity. Author manuscript; Available in PMC 2026 Sep 4. (PMC7619444; doi:10.1016/j.immuni.2024.04.018)
Supplement: Supplementary file 3 [file EMS217793-supplement-Resource_file.docx]

**KEY RESOURCES TABLE**

The table highlights the reagents, genetically modified organisms and strains, cell lines, software, instrumentation, and source data **essential** to reproduce results presented in the manuscript. Depending on the nature of the study, this may include standard laboratory materials (i.e., food chow for metabolism studies, support material for catalysis studies), but the table is **not** meant to be a comprehensive list of all materials and resources used (e.g., essential chemicals such as standard solvents, SDS, sucrose, or standard culture media do not need to be listed in the table). **Items in the table must also be reported in the method details section within the context of their use.** To maximize readability, the number of **oligonucleotides and RNA sequences** that may be listed in the table is restricted to no more than 10 each. If there are more than 10 oligonucleotides or RNA sequences to report, please provide this information as a supplementary document and reference the file (e.g., See Table S1 for XX) in the key resources table.

***Please note that ALL references cited in the key resources table must be included in the main references list.*** Please report the information as follows:

- **REAGENT or RESOURCE:** Provide the full descriptive name of the item so that it can be identified and linked with its description in the manuscript (e.g., provide version number for software, host source for antibody, strain name). In the experimental models section (applicable only to experimental life science studies), please include all models used in the paper and describe each line/strain as: model organism: name used for strain/line in paper: genotype. (i.e., Mouse: OXTR^fl/fl^: B6.129(SJL)-Oxtr^tm1.1Wsy/J^). In the biological samples section (applicable only to experimental life science studies), please list all samples obtained from commercial sources or biological repositories. Please note that software mentioned in the methods details or data and code availability section needs to also be included in the table. See the sample tables at the end of this document for examples of how to report reagents.
- **SOURCE:** Report the company, manufacturer, or individual that provided the item or where the item can be obtained (e.g., stock center or repository). For materials distributed by Addgene, please cite the article describing the plasmid and include “Addgene” as part of the identifier. If an item is from another lab, please include the name of the principal investigator and a citation if it has been previously published. If the material is being reported for the first time in the current paper, please indicate as “this paper.” For software, please provide the company name if it is commercially available or cite the paper in which it has been initially described.
- **IDENTIFIER:** Include catalog numbers (entered in the column as “Cat#” followed by the number, e.g., Cat#3879S). Where available, please include unique entities such as [RRIDs](https://www.force11.org/group/resource-identification-initiative), Model Organism Database numbers, accession numbers, and PDB, CAS, or CCDC IDs. For antibodies, if applicable and available, please also include the lot number or clone identity. For software or data resources, please include the URL where the resource can be downloaded. Please ensure accuracy of the identifiers, as they are essential for generation of hyperlinks to external sources when available. Please see the Elsevier [list of data repositories](https://www.elsevier.com/authors/author-resources/research-data/data-base-linking) with automated bidirectional linking for details. When listing more than one identifier for the same item, use semicolons to separate them (e.g., Cat#3879S; RRID: AB_2255011). If an identifier is not available, please enter “N/A” in the column.
  - ***A NOTE ABOUT RRIDs:*** We highly recommend using RRIDs as the identifier (in particular for antibodies and organisms but also for software tools and databases). For more details on how to obtain or generate an RRID for existing or newly generated resources, please [visit the RII](https://www.force11.org/group/resource-identification-initiative) or [search for RRIDs](https://scicrunch.org/resources).

Please use the empty table that follows to organize the information in the sections defined by the subheading, skipping sections not relevant to your study. Please do not add subheadings. To add a row, place the cursor at the end of the row above where you would like to add the row, just outside the right border of the table. Then press the ENTER key to add the row. Please delete empty rows. Each entry must be on a separate row; do not list multiple items in a single table cell. Please see the sample tables at the end of this document for relevant examples in the life and physical sciences of how reagents and instrumentation should be cited.

***TABLE FOR AUTHOR TO COMPLETE***

*Please upload the completed table as a separate document.* ***Please do not add subheadings to the key resources table.*** *If you wish to make an entry that does not fall into one of the subheadings below, please contact your handling editor.* ***Any subheadings not relevant to your study can be skipped.*** *(****NOTE:*** *References within the KRT should be in numbered style rather than Harvard.)*

**Key resources table**

| **REAGENT or RESOURCE** | **SOURCE** | **IDENTIFIER** |
| --- | --- | --- |
| **Antibodies** | | |
| Rabbit polyclonal anti Dclk1 | Abcam | Cat# Ab31704, RRID:AB_873537 |
| Sheep polyclonal to Dclk1 | R&D Systems | Cat# AF7138 RRID:AB_10973467 |
| Rabbit monoclonal [EPR16651] to Gata3 | Abcam | Cat# Ab199428, RRID:AB_2819013 |
| Rabbit polyclonal to Retnlb | Antibodies online | Cat# ABIN465494 |
| Mouse monoclonal to Insm1 | Santa Cruz | Cat# sc-271408, RRID:AB_10607955 |
| Rabbit polyclonal to Lysozyme | Dako | Cat# A0099 |
| Rabbit polyclonal to Granzyme-B antibody | Abcam | Cat# Ab4059, RRID:AB_304251 |
| Rat monoclonal to Cd45 | Novus | Cat# NB100-77417, RRID:AB_1083776 |
| Rat monoclonal to MCPT-1 (mMCP-1) Antibody | eBioscience/ThermoFisher | Cat# 14-5503-82, RRID:AB_10854869 |
| Goat polyclonal to C-Kit antibody | R&D Systems | Cat# AF1356, RRID:AB_354750 |
| Rabbit polyclonal to Collagen V antibody | Gift From Patricia Simon Assmann | N/A |
| Rat anti mouse Siglec-F, PE-conjugated | BD pharmigen | Cat# 552126, RRID:AB_394341 |
| Rat anti mouse EpCam, APC-conjugated | eBioscience/ThermoFisher | Cat# 17-5791-82,  RRID:AB_2716944 |
| Rabbit monoclonal to 5-Lipoxygenase Antibody (5Alox) | Novus | Cat# NBP3-161-50 |
| Goat polyclonal to FLAP (Alox5Ap) Antibody | Novus | Cat# NB300-891, RRID:AB_2227081 |
| Rabbit polyclonal to cyclooxygenase 2 antibody | Abcam | Cat# Ab225273 |
| Rabbit polyclonal to Prostaglandin D Synthase (hematopoietic-type) Antibody | Cayman chemical | Cat# 160013,  RRID:AB_10080037 |
| Rabbit monoclonal to CD63 antibody | Abcam | Cat# Ab217345,  RRID:AB_2754982 |
| Mouse monoclonal to Estrogen Receptor alpha antibody | Santa Cruz | Cat# Sc-8002,  RRID:AB_627558 |
| Rabbit Monoclonal Anti-TBX21 (T-bet) | Clinisciences | Cat# AC-0240A |
| Rabbit Anti-Rorgt (clone EPR20006) | Abcam | Cat# ab207082, RRID:AB_2889310 |
| Rat Anti-mCD45-FITC (clone 30-F11) | BD Biosciences | Cat# 553080, RRID:AB_394610 |
| Rat Anti-mCD45-APC (clone 30-F11) | BD Biosciences | Cat# 559864, RRID:AB_398672 |
| Rat Anti-mCD45- APC-eF780 (clone 30-F11) | eBioscience/ThermoFisher | Cat#47-0451-80  RRID: AB_1548790 |
| Rat Anti-mCD19- BV650 (clone 1D3) | BD Biosciences | Cat# 563235,  RRID:AB_273808 |
| Rat Anti-mCD19- V450 (clone 1D3) | BD Biosciences | Cat# 560375  RRID:AB_164526 |
| Hamster Anti-CD11c- PE (clone HL3) | BD Biosciences | Cat#557401,  RRID:AB_396684 |
| Rat Anti-mCD11b-BV605 (clone M1/70) | BD Biosciences | Cat# 563015, RRID:AB_2737951 |
| Rat Anti-mCD4- BV711 (clone RM4-5) | BD Biosciences | Cat#563726,  RRID: [AB_2738389](http://antibodyregistry.org/AB_1272179) |
| Rat Anti-mCD4- BV786 (clone RM4-5) | BD Biosciences | Cat# 563727,  RRID:AB_2728707 |
| Hamster Anti-mTCRβ- FITC (clone H57-597) | BD Biosciences | Cat# 553170, RRID:AB_394682 |
| Hamster Anti-mTCRβ- AF594 (clone H57-597) | Biolegend | Cat# 109238,  RRID: [AB_2563324](http://antibodyregistry.org/AB_2563324) |
| Hamster Anti-mTCRβ- V450 (clone H57-597) | BD Biosciences | Cat# 560706, RRID:AB_1727576 |
| Rat Anti-mCD8α- APC-R700 (clone 53-6.7) | BD Biosciences | Cat#564983,  RRID: [AB_2739032](http://antibodyregistry.org/AB_2739032) |
| Rat Anti-mCD8b- PE (clone H35-17.2) | BD Biosciences | Cat# 550798, RRID:AB_393887 |
| Rat Anti-mCD117- BV711 (clone2B8) | BD Biosciences | Cat# 563160, RRID:AB_272251 |
| Rat Anti-mCD117- PECF594 (clone2B8) | BD Biosciences | Cat# 562417, RRID:AB_11154233 |
| Rat Anti-mSca-1- FITC (clone D7) | BD Biosciences | Cat# 557405,  RRID:AB_396688 |
| Rat Anti-mCD127- PE (clone A7R34) | eBioscience/ThermoFisher | Cat# 12-1271-82, RRID:AB_465844 |
| Hamster Anti-mKLRG1- PeCy7 (clone 2F1) | eBioscience/ThermoFisher | Cat# 25-5893-82, RRID:AB_1518768 |
| Anti-mGATA3- V450 (clone L50-823) | BD Biosciences | Cat# 563349,  RRID:AB_2738152 |
| Mouse Anti-mRoRγt- BV650 (clone Q31378) | BD Biosciences | Cat# 564722, RRID:AB_2738915 |
| Mouse Anti-mTbet- PE (clone 4B10) | eBioscience/ThermoFisher | Cat#12-5825-82,  RRID:AB_925761 |
| Rat Anti-mF4/80- PeCy7 (clone BM8) | eBioscience/ThermoFisher | Cat# 25-4801-82,  RRID:AB_469653 |
| Rat Anti-mLy6C- V450 (clone HK1.4) | eBioscience/ThermoFisher | Cat# 48-5932-82, RRID:AB_10805519 |
| Rat Anti-mLy6G- PE-CF594 (clone 1A8) | BD Biosciences | Cat# 562700, RRID:AB_2737730 |
| Hamster Anti-mFceRIα - APC (clone MAR-1) | eBioscience/ThermoFisher | Cat# 17-5898-80,  RRID:AB_10717073 |
| Live/Dead V506 | eBioscience/ThermoFisher | Cat#65-0866-14 |
| Donkey Anti-Rabbit IgG H&L (Alexa Fluor® 555) | Abcam | Cat# ab150074  RRID:AB_2715537 |
| Donkey Anti-Rabbit IgG H&L (Alexa Fluor® 647) | Abcam | Cat# ab150075  RRID:AB_2752244 |
| Donkey Anti-Rat IgG H&L (Alexa Fluor® 647) preadsorbed | Abcam | Cat# ab150155  RRID:AB_2813835 |
| Donkey Anti-Rat IgG H&L (Alexa Fluor® 488) preadsorbed | Abcam | Cat# ab150153  RRID:AB_2737355 |
| Donkey Anti-Mouse IgG H&L (Alexa Fluor® 555) | Abcam | Cat# ab150106  RRID:AB_2857373 |
| Donkey Anti-Mouse IgG H&L (Alexa Fluor® 488) | Abcam | Cat# ab150105  RRID:AB_2732856 |
| Donkey Anti-Sheep IgG H&L (Alexa Fluor® 488) | Abcam | Cat# ab150177  RRID:AB_2801320 |
| Donkey Anti-Goat IgG H&L (Alexa Fluor® 555) | Abcam | Cat# ab150130  RRID:AB_2715537 |
|  |  |  |
| **Bacterial and virus strains** |  |  |
|  |  |  |
| **Biological samples** |  |  |
|  |  |  |
| **Chemicals, peptides, and recombinant proteins** |  |  |
| Neostigmine Bromide | Sigma-Aldrich | Cat# N2001 |
| Acetylcholine chloride | Sigma-Aldrich | Cat# A6625 |
| Atropine | Sigma-Aldrich | Cat# A0132 |
| Mecamylamine hydrochloride | Sigma-Aldrich | M9020 |
| RPMI culture medium | Life Technologies | Cat# 21875-034 |
| DMEM culture medium | Life Technologies | Cat# 21969-035 |
| FBS | Sigma-Aldrich | Cat# F7524 |
| Dispase | Corning | Cat# 354235 |
| DNAse 1 | Sigma-Aldrich | Cat# 11284932001 |
| 7-aminoactinomycin D | Life Technologies | Cat# A1310 |
| HBSS 10X w/o Ca2+ / Mg2+ | GIBCO | Cat# 14185-045 |
| HBSS 1X avec Ca2+ / Mg2+ | GIBCO | Cat# 14025-050 |
| PBS 1X (Ca2+ et Mg2+ free) | Eurobio | Cat# CS1PB01-01 |
| LiberaseTM | Roche | Cat# 5401119001 |
| EDTA | Sigma-Aldrich | Cat# 03690-100 |
| DTT | Sigma-Aldrich | Cat# 43816-50 |
| Vetflurane 1000mg/g | Virbac | GTIN 03597132002653 |
| Buprecare 0.3mg/ml | Axience | GTIN 03760087151244 |
| Tamoxifene | Sigma-Aldrich | Cat# T5648 |
| Laocaine 16mg | MSD | GTIN : 05017363520132 |
| Trizol reagent | Thermo Fisher | Cat# 15596026 |
| Buprenorphine | Sigma-Aldrich | Cat#B9275 |
| Hematoxylin Solution, Gill No. 3 | Sigma-Aldrich | Cat#GHS316 |
| SIGMAFAST™ 3,3′-Diaminobenzidine tablets | Sigma-Aldrich | Cat# D4293 |
| N-Histofine | Nichirei Biosciences | 414151F |
| **Critical commercial assays** |  |  |
| ATPLite kit | Perkin Elmer | Cat# 6016943 |
| RNeasy Micro Kit | Qiagen | ID: 74004 |
| RNeasy Mini Kit | Qiagen | ID: 74104 |
| Transcriptor First Strand cDNA Synthesis Kit | Roche lifescience | Cat# 04896866001 |
| LightCycler® 480 SYBR Green I Master | Roche lifescience | Cat# 04887352001 |
| RNAscope™ Multiplex Fluorescent V2 Assay | ACD a biotechne brand | Cat# 323270 |
| Fix/perm kit FOXP3/transcription factor staining buffer set | eBioscience/ThermoFisher | Cat# 00-5523-00 |
| Arcturus RiboAmp Plus kit | Thermo Fisher | Cat# KIT0501 |
| **Deposited data** |  |  |
|  |  |  |
| **Experimental models: Cell lines** |  |  |
|  |  |  |
| **Experimental models: Organisms/strains** |  |  |
| Mouse strain Chat^tm1Mlt^ | Lecomte et al. ^(22)^ | Provided by S. Berrard |
| Mouse strain Tg(Vil1-cre/ERT2)23Syr | El Marjour et al. ^(23)^ | Provided by S. Robine |
| Mouse strain Chat^LoxP/LoxP^; VillinCre^ERT2/+^ | This study | N/A |
| *Heligmosomoides polygyrus* | Provided by R. Maizels | Provided by R. Maizels |
| **Oligonucleotides** |  |  |
| *Chat* | Forward TTTGCAGCCAGCCTCATCTC  Reverse TATGGCCAGGAAGCCGGTAT | Eurofins |
| *VAChT* | Forward GGCCTCGCTACCCCACAGAA  Reverse CCCAGGCCAATAAGCAGCGG | Eurofins |
| *Pou2f3* | Forward GAGGGAATGATGAGCCCACT  Reverse GTGAAGCCTAGCTTAATGCGTC | Eurofins |
| *Alox5* | Forward TCTTCCTGGCACGACTTTGCTG  Reverse GCAGCCATTCAGGAACTGGTAG | Eurofins |
| *Alox5ap* | Forward GTTCTTTGCCCACAAGGTGGAG  Reverse TGCAGTCCAGAGTACCACAAGG | Eurofins |
| *Ptgs1* | Forward GAATGCCACCTTCATCCGAGAAG  Reverse GCTCACATTGGAGAAGGACTCC | Eurofins |
| *Ptgs2* | Forward TCAATGAGTACCGCAAACGC  Reverse AGGGTACAGTTCCATGACATCG | Eurofins |
| *Hpgds* | Forward GAATAGAACAAGCTGACTGGC  Reverse AGCCAAATCTGTGTTTTTGG | Eurofins |
| *Ltc4s* | Forward CCTACAGGTGATCTCTGCACGA  Reverse TGGCGAGGAACAGCGGAAAGTA | Eurofins |
| *Sucnr1* | Forward CCATCTCTGACTTTGCTTTCCTG  Reverse GTGTAGAGGTTGGTGTGAAGCAC | Eurofins |
| *Dclk1* | Forward CAGCCTGGACGAGCTGGTGG  Reverse TGACCAGTTGGGGTTCACAT | Eurofins |
| *Il25* | Forward CCTGTCAGGCAGGGGTAGTA  Reverse CCAAGAATGCAACAGCCTG | Eurofins |
| *Villin* | Forward: CAAGCCTGGCTCGACGGCC  Reverse : CGCGAACATCTTCAGGTTCT | Eurofins |
| *Chat* (exon8) | Forward: CCCGTTTTCCTTCCAGCCAA  Reverse : CGCGAACATCTTCAGGTTCT | Eurofins |
| *Gapdh* | Forward GGAGCGAGACCCCACTAACA  Reverse ACATACTCAGCACCGGCCTC | Eurofins |
| *Hprt* | Forward GCAGTACAGCCCCAAAATGG  Reverse GGTCCTTTTCACCAGCAAGCT | Eurofins |
| **Recombinant DNA** |  |  |
|  |  |  |
| **Software and algorithms** |  |  |
| NdpView.V2 | Hamamatsu Photonics | https://www.hamamatsu.com |
| Zen blue edition. V3.2 | Zeiss | https://www.zeiss.com/microscopy/fr/produits/logiciel/zeiss-zen.html |
| Photoshop CS6. V13.0 | Adobe | https://www.adobe.com/fr/products/photoshop.html |
| Prism10.1 | GraphPad | https://www.graphpad.com |
| LightCycler 480 V1.5 | Roche | https://lightcycler-software.software.informer.com |
| ImageJ. V1.8 | Wayne Rasband (NIH) | https://imagej.net/ij/download.html |
| FlowJo, LLC V9 | Tree Star; Ashland, OR | https://www.flowjo.com/ |
| Biorender | BioRENDER | https://www.biorender.com/ |
| **Other** |  |  |
| RNAscope™ Probe- Mm-Chat-C2 | Advanced Cell Diagnostics | Cat# 408731-C2 |
| RNAscope™ Probe- Mm-Sucnr1-C3 | Advanced Cell Diagnostics | Cat# 437721-C3 |
| RNAscope™ Probe- Mm-Ltc4s-C3 | Advanced Cell Diagnostics | Cat# 1046751-C3 |
